# Host genotype-driven shifts in the *Medicago* seed microbiome reveal domestication-linked diversity loss in lucerne (*Medicago sativa*)

**DOI:** 10.3389/fmicb.2025.1660250

**Published:** 2025-11-04

**Authors:** Shenali Subodha Herath Dissanayakalage, Jatinder Kaur, Ross C. Mann, Timothy I. Sawbridge

**Affiliations:** ^1^Department of Energy, Environment and Climate Action, Agriculture Victoria, AgriBio, Centre for AgriBioscience, Bundoora, VIC, Australia; ^2^School of Applied Systems Biology, La Trobe University, Bundoora, VIC, Australia

**Keywords:** alfalfa, crop wild relatives, seed bacterial microbiome, core microbiome, microbial diversity, microbiome profiling, microbial isolations, culturability

## Abstract

Seed microbiomes represent a critical yet underexplored dimension of plant-associated microbial communities, with potential to enhance crop resilience and sustainability. While plant microbiomes have gained prominence, the diversity and composition of seed-associated bacteria—especially across wild and domesticated lineages—remain poorly characterised. Here, we profiled the bacterial seed microbiome of lucerne (*Medicago sativa* L.) and its crop wild relatives using an integrative approach combining amplicon sequencing, culture-based recovery, and whole-genome analysis of representative isolates. Amplicon profiling revealed a conserved core microbiome across all accessions, alongside host-genotype-specific patterns and markedly higher bacterial diversity in wild relatives. Culture-based methods recovered over half of the abundant amplicon sequence variants (ASVs), validating the representativeness of the isolate library. The whole genome sequencing of selected isolates uncovered substantial intra-species variation, including genomically distinct strains within the same species. Core taxa such as *Pantoea*, *Paenibacillus*, and *Pseudomonas* were consistently recovered, while several genera enriched in wild relatives—*Massilia*, *Duganella*, *Sphingomonas*—were absent or rare in domesticated lines. Comparative microbiome analysis revealed that domestication has reduced both taxonomic richness and microbial variability in the lucerne seed microbiome. The dominance of conserved taxa alongside the exclusion of wild-enriched groups suggests that breeding history influences microbial assembly and may constrain microbiome function. The consistent presence of core taxa across accessions is consistent with the possibility that, vertical transmission, together with host genotype, contributes to seed microbiome structure. By linking plant genotype with seed microbiome composition and culturability, this study provides a high-resolution view of seed microbial assembly shaped by evolutionary history. The resulting culture-based microbial resource, supported by genome-level characterisation of representative taxa, offers a robust foundation for microbiome-informed strategies in lucerne breeding and pasture improvement.

## 1 Introduction

Global food security amid rising environmental and economic pressures demand a shift toward more resilient and sustainable cropping systems. Plant-associated microbial communities are increasingly recognised as key contributors to crop health, supporting nutrient acquisition, stress tolerance, and disease suppression (Compant et al., 2025; Joshi et al., 2025). Although the concept of beneficial plant–microbe interactions date back to the early 20th century, when Lorenz Hiltner first proposed the idea of “rhizosphere effect” (Hartmann et al., 2008), recent advances have revealed the complexity and functional importance of microbial consortia inhabiting the rhizosphere, endosphere, and phyllosphere. These communities influence host function through multiple pathways, such as nitrogen (N) fixation, phytohormone production, phosphorus (P) solubilisation, and iron sequestration (Vorholt, 2012; Berg et al., 2014; Mabood et al., 2014; Reinhold-Hurek et al., 2015; Tkacz and Poole, 2015). In parallel, microbial inoculants are emerging as eco-friendly alternatives to agrochemicals with several strains, such as *Azospirillum* (Mazospirflo-2^®^, Soilgro), *Azotobacter* (Bio-N™, Agriculture Solutions) and *Bacillus megatherium* (Symbion-P^®^) already commercialised for agricultural use (Owen et al., 2015; Elsayed et al., 2020).

Seeds form a distinct microbial niche with important implications for vertical transmission and early plant development. The seed-associated microbiota, comprising both epiphytic and endophytic populations colonise the seed coat, storage tissues, and embryo, and is either inherited or acquired from the environment (Hashidoko, 2005; Links et al., 2014; Truyens et al., 2015). Vertically transmitted microbes can shape microbial assembly in seedlings, influencing germination, growth, and responses to stress (Puente et al., 2009; Oukala et al., 2021; Abdelfattah et al., 2023). However, conventional culture-based methods often underrepresent the full taxonomic and functional diversity of seed microbiota (Rahman et al., 2018; Chandel et al., 2022b), limiting ecological insight and translational potential.

Similar to other plant compartments, seeds host a “core microbiome,” defined as microbial taxa that are consistently associated with the host across environments and genotypes (Simonin et al., 2022). These core members are shaped by host selection and environmental filtering, and are believed to perform essential ecological functions (Turnbaugh et al., 2007; Shade and Handelsman, 2012; Vandenkoornhuyse et al., 2015; Astudillo-García et al., 2017). Across various plant species, seed microbiota is typically dominated by Proteobacteria, Firmicutes, Actinobacteria and Bacteroidetes. Genera such as *Pantoea*, *Enterobacter*, and *Pseudomonas* are frequently identified in the seeds of barley (*Hordeum vulgare*), maize (*Zea mays*), rice (*Oryza sativa*), and sunflower (*Helianthus annuus*) (Johnston-Monje and Raizada, 2011; Leff et al., 2017; Rahman et al., 2018; Eyre et al., 2019; Hardoim, 2019; Johnston-Monje et al., 2021).

Domestication has significantly altered plant phenotypes, genomes, and associated microbial communities. One of the major consequences is the reduction in genetic diversity, as observed in rice, wheat (*Triticum aesitivum*), and common bean (*Phaseolus vulgaris*) (Haudry et al., 2007; Ram et al., 2007; Bitocchi et al., 2013). In maize, certain core seed-associated bacteria, including *Paenibacillus*, *Enterobacter*, *Methylobacterium*, *Pantoea* and *Pseudomonas* have persisted post-domestication (Johnston-Monje and Raizada, 2011), yet wild relatives of crops such as rice, peas (*Pisum sativum*) and soybean (*Glycine max*) often retain greater microbial richness (Shi et al., 2019). These patterns suggest that domestication may constrain microbiome diversity and modify plant–microbe interactions. Although such trends are well documented in cereals (Kim et al., 2020; Abdullaeva et al., 2021), seed-level studies in legumes remain limited, underscoring the potential of wild relatives as reservoirs of microbial diversity absent in elite cultivars.

Lucerne or alfalfa (*Medicago sativa* L.; hereafter referred to as domesticated lucerne) is one of the earliest domesticated legumes (Gault et al., 1995; Bouton, 2012), is currently the most widely cultivated perennial pasture legumes globally and in Australia, valued for its role in sustainable cropping systems through N fixation, soil improvement, and weed and disease suppression (Humphries and Auricht, 2001; Latta et al., 2002). In contrast, *Medicago* crop wild relatives (hereafter referred to as *Medicago* CWRs) shaped by diverse natural environments, possess broader adaptive traits and potentially richer microbial communities (Annicchiarico et al., 2015). Domesticated lucerne has undergone an estimated 30% reduction in genetic diversity (Muller et al., 2006), with emerging evidence suggests a similar narrowing of its microbiome. For instance, *M. polymorpha*, a wild *Medicago* species, harbours plant growth-promoting (PGP) bacteria underrepresented in domesticated lucerne varieties (Martínez-Hidalgo et al., 2022). Yet, the seed-associated microbiome of lucerne and its CWRs remains underexplored, particularly in terms of diversity, culturability, and translational potential. As vertically transmitted microbes influence early plant development, elucidating these communities could support future strategies for microbial inoculant development and microbiome-informed breeding (Shade et al., 2017; Abdelfattah et al., 2023).

This study characterises and compares the seed-associated bacterial communities of domesticated lucerne and selected *Medicago* CWRs to assess whether wild genotypes harbour more diverse and compositionally distinct microbiota. Eighteen domesticated lucerne accessions, sourced from commercial seed suppliers across Australia, and eighteen CWR accessions from Libya and Russia, were obtained from the Australian Pastures Genebank (APG). We hypothesised that CWR seeds harbor more diverse and compositionally distinct microbial communities than domesticated lucerne. To retain both epiphytic and endophytic microbes, seeds were rinsed but not surface-sterilised. Culturable bacteria were isolated and identified using conventional microbiological techniques and Sanger sequencing. In parallel, amplicon-based 16S rRNA gene profiling was used to characterise broader community structure, and a subset of isolates underwent whole-genome sequencing (WGS). Together, these approaches provide integrated insights into the structure and culturability of *Medicago* seed microbiome, laying the groundwork for microbiome-informed lucerne improvement strategies.

## 2 Materials and methods

### 2.1 *Medicago* seed collection, washing, and germination

**FIGURE 1:**
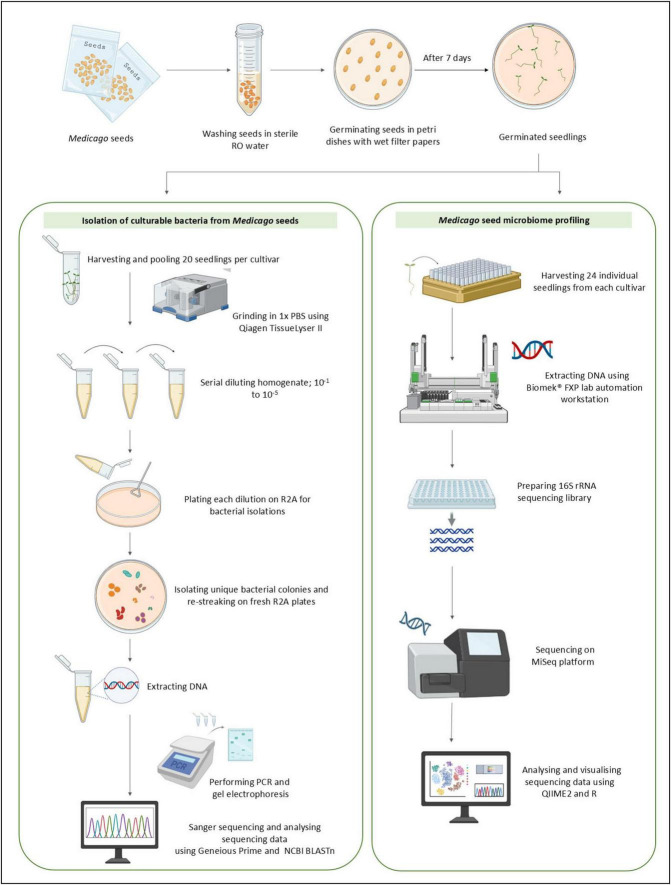
Dual pipeline for isolating culturable bacteria and profiling the bacterial microbiome of *Medicago* seeds. Washed seeds were germinated and processed via two complementary approaches. The culture-dependent workflow involved bacterial isolation and taxonomic identification using PCR and Sanger sequencing. The culture-independent workflow involved direct DNA extraction, 16S rRNA gene amplicon sequencing, and microbial community profiling.

Seeds from eighteen domesticated lucerne cultivars and eighteen *Medicago* CWR accessions were sourced from commercial seed companies across Australia and the APG, respectively (Table 1). Although APG accessions trace back to international origins (e.g., Libya, Russia), the seed lots provided for this study were regenerated under Australian conditions prior to distribution. The seeds were stored at 4 °C in a Controlled Environment Room (CER) at AgriBio Institute, Bundoora, Victoria, Australia. For each accession, 0.2 g of seeds (approximately 420 seeds/g) were washed by rinsing four times with autoclaved reverse-osmosis (RO) water (Figure 1). During the final rinse, seeds were incubated for 4 min at room temperature to facilitate the removal of loosely associated bacteria from the seed coat. A 100 μL aliquot of the final wash was plated onto Reasoner’s 2A (R2A; Oxoid or Amyl Media, Australia) and plates were incubated at room temperature for 7 days to confirm the absence of culturable bacteria. After washing, seeds were placed on sterile, moist filter paper in 90 mm sealed petri dishes for germination. Filter papers were moistened with 3 mL of sterile RO water. For each accession, fifteen seeds were placed per petri dish, and five replicate plates were prepared. Germination was conducted at room temperature for 7 days.

**TABLE 1:** Domesticated lucerne and *Medicago* CWR seed accessions used in this study.

No.	Group	*Medicago* seed accession/cultivar	Source/Company	Species	Accession code	Origin	Germination %
1	Commercial *Medicago* accessions	Sequel	Green Harvest	*Medicago sativa*	Sq	Australia	100.0%
2		Hunter River	Green Harvest	*Medicago sativa*	HR		98.6%
3		Trifecta	Eden seeds	*Medicago sativa*	Ed		75.0%
4		Aurora	Australian Wheatgrass	*Medicago sativa*	Au		100.0%
5		Siriver	Healthforce	*Medicago sativa*	Sv		95.0%
6		Ryno 6	AGF Seeds	*Medicago sativa*	R6		94.3%
7		SF Force 5	Seed Force	*Medicago sativa*	F5		93.6%
8		SARDI 7 Series 2	Barenbrug	*Medicago sativa*	SS		92.1%
9		SARDI 10 Series 2	Barenbrug	*Medicago sativa*	ST		94.7%
10		SARDI Grazer	Barenbrug	*Medicago sativa*	SG		92.6%
11		Genesis	Synergy Seeds	*Medicago sativa*	GN		93.8%
12		Silverado	Upper Murray seeds	*Medicago sativa*	SL		95.2%
13		Mr Fothergills	Sprouts Alive	*Medicago sativa*	Ft		58.6%
14		Magna 959 (mature seeds)	A farm, South Australia	*Medicago sativa*	MM		22.0%
15		Magna 959 (young seeds)	A farm, South Australia	*Medicago sativa*	MY		15.0%
16		SF 714	Seed Force	*Medicago sativa*	SF714		80.3%
17		SF 730	Seed Force	*Medicago sativa*	SF730		82.4%
18		SF 914	Seed Force	*Medicago sativa*	SF914		80.9%
19	*Medicago* CWR accessions	APG 6032	Australian Pastures Genebank (APG)	*Medicago sativa* subsp. *falcata*	FL32	Russia	90.2%
20		APG 6039		*Medicago sativa* subsp. *falcata*	FL39		92.1%
21		APG 6925		*Medicago sativa* subsp. *falcata*	FL25		90.9%
22		APG 20535		*Medicago littoralis* var. *littoralis*	LTV_535	Libya	94.7%
23		APG 21384		*Medicago littoralis* var. *littoralis*	LTV_384		95.0%
24		APG 21559		*Medicago littoralis* var. *littoralis*	LTV_559		94.2%
25		APG 32892		*Medicago littoralis* var. *littoralis*	LTV_892		95.9%
26		APG 21164		*Medicago laciniata*	LA164		96.0%
27		APG 21177		*Medicago laciniata*	LA177		96.8%
28		APG 20841		*Medicago laciniata*	LA841		94.9%
29		APG 21700		*Medicago laciniata*	LA700		95.6%
30		APG 20935		*Medicago truncatula*	TR935		93.5%
31		APG 21758		*Medicago truncatula*	TR758		94.2%
32		APG 21771		*Medicago truncatula*	TR771		94.9%
33		APG 21177		*Medicago littoralis*	LT177		82.3%
34		APG 21198		*Medicago littoralis*	LT198		74.1%
35		APG 21232		*Medicago littoralis*	LT232		78.3%
36		APG 21235		*Medicago littoralis*	LT235		75.3%

### 2.2 Isolation of culturable bacteria from seeds

To isolate seed-associated bacteria, twenty healthy seedlings per cultivar were harvested after 7 days of germination and suspended in 300 μL of sterile 1 × phosphate-buffered saline (PBS). Samples were homogenised using a Qiagen TissueLyser II (2 × 30 s at 25 Hz). The resulting homogenate from each cultivar was serially diluted (1:10, 100 μL in 900 μL) and plated onto R2A to isolate distinct bacterial colonies from 10^–2^ to 10^–5^ dilutions. Pure cultures were preserved in nutrient broth (NB) supplemented with 20% (v/v) glycerol and stored at -80 °C until further use (Herath Dissanayakalage et al., 2025a).

### 2.3 Molecular identification of culturable bacterial endophytes

Direct 16S colony PCR was performed on all bacterial isolates. For those that did not yield PCR products, genomic DNA was extracted and used as a template. Single colonies (1–2 mm in diameter) were picked using a sterile needle or pipette tip and suspended in 50 μL of nuclease-free water. Samples were incubated at 99 °C for 10 min, and 2 μL of the resulting supernatant was used directly as template DNA.

PCR reactions (25 μL total volume) contained 12.5 μL of OneTaq Hot Start 2 × Master Mix with standard buffer (M0484, Promega, Madison, WI, USA), 1.0 μL of each primer (10 pmol/μL; 27F: AGAGTTTGATCMTGGCTCAG and 1492R: GGTTACCTTGTTACGACTT), and Milli-Q water to volume. A no-template control was included in each run. The thermocycling conditions were as follows: initial denaturation at 95 °C for 1 min; 35 cycles of denaturation at 94 °C for 30 s, annealing at 55 °C for 30 s, and elongation at 72 °C for 1 min; followed by a final extension at 72 °C for 5 min.

Genomic DNA for non-amplifying isolates was extracted using Wizard^®^ Genomic DNA Purification Kit (A1120, Promega, Madison, WI, United States) with minor modifications to the manufacturer’s protocol. Overnight liquid cultures (1 mL) were centrifuged twice at 13,000–16,000 × *g* for 2 min to maximise cell recovery. The samples were incubated at -20 °C for 10 min instead of on ice to enhance precipitation of most of the proteins bound to DNA. DNA pellets were rehydrated in 50 μL rehydration solution at 65 °C for 10 min, followed by overnight incubation at 4 °C. DNA concentration was measured using a NanoDrop 2000/2000c spectrophotometer (Thermo Scientific, Waltham, MA, USA). The PCR reaction mixture and thermocycling conditions were identical to those described above, except 5 μL of purified DNA was used as the template.

#### 2.3.1 Sanger sequencing and taxonomic identification

Amplified 16S rRNA gene products (∼ 1,400bp), normalised to a concentration of 50 ng/μL, were separated by electrophoresis at 100 V in a 1.5% agarose gel containing SYBR safe DNA gel stain (0.05 μL/mL) in 1 × TBE running buffer. PCR bands were visualised under UV light (360 nm) using a ChemiDoc MP imaging system (Bio-Rad) to confirm amplification success. Amplified products were then submitted to Macrogen, Inc., (Seoul, South Korea) for Sanger sequencing. Raw sequence data were initially analysed using NCBI BLASTn for preliminary taxonomic identification.

Reads were quality-trimmed in Geneious Prime (version 2020.0.2; Biomatters Ltd., Auckland, New Zealand), using default parameters to remove low quality bases (Phred score < 20) and ambiguous ends. The reverse read was reverse-complemented prior to alignment. Pairwise alignment of forward and reverse reads was performed using the Geneious alignment tool with default parameters to generate consensus sequences. Taxonomic identity was assigned using NCBI BLASTn based on ≥97% sequence similarity to type strains in the 16S rRNA reference database. All sequences were submitted to NCBI under BioProject accession PRJNA1180717.

#### 2.3.2 Genome sequencing using Oxford Nanopore Technologies (ONT)

Long-read sequencing was performed on selected bacterial isolates using ONT. Genomic libraries were prepared using the ONT ligation sequencing kit (SQK-LSK109; Kit 9 chemistry; ONT, Oxford, United Kingdom), with minor protocol modifications to improve DNA recovery. Sequencing was conducted on a MinION Mk1B device (MIN-101B) using R9.4.1 flow cells. Raw FAST5 signal files were base called using the Guppy command-line tool (v5.0.11) (Wick et al., 2019). Demultiplexing and adapter trimming were performed using the guppy_barcoder module, and high-quality FASTQ files were generated. Sequence quality was assessed using FastQC prior to downstream analysis.

#### 2.3.3 Genome assembly, taxonomic classification, and comparative analysis

Long-read genome assemblies were generated using Trycycler (Wick et al., 2021). Sequencing reads were first subsampled into multiple subsets and assembled independently using Flye (Freire et al., 2022). The resulting assemblies were then reconciled into consensus genomes using Trycycler’s consensus workflow. Assembled genomes were taxonomically classified using Kraken2 (Wood et al., 2019), with a custom database built from all complete bacterial reference genomes available in NCBI as of March 2023.

To assess genome-level relatedness, average nucleotide identity (ANI) was calculated as a measure of overall genome relatedness index (OGRI) (Richter and Rosselló-Móra, 2009). Species-level classification was determined using an ANI threshold of ≥95%, consistent with established standards for prokaryotic species delineation. Pairwise ANI values were computed using a Perl script, and species-level comparisons among closely related isolates were performed using minimap2 (Li, 2018) for genome alignment and identity calculation.

### 2.4 *Medicago* microbiome profiling

#### 2.4.1 DNA extraction, 16S amplicon library preparation, and sequencing

Total DNA was extracted from individual *Medicago* seedlings (*n* = 24 seedlings per cultivar) using the QIAGEN MagAttract 96 DNA Plant Core Kit (Qiagen^®^, Hilden, Germany), with minor modifications to the manufacturer’s protocol. Extractions were carried out on a Biomek^®^ FXP lab automation workstation operated via Biomek^®^ software v4.1 and Gen5 (v2.08) (Beckman Coulter, Brea, CA USA). Amplicon libraries targeting the V4 region of the 16S rRNA gene were prepared using a two-step PCR with PNA blockers, indexed with Nextera XT dual indices, and sequenced on the Illumina MiSeq platform (2 × 300 bp). Detailed protocols, including thermocycling conditions and reagent concentrations, are provided in Supplementary Section 1.

#### 2.4.2 MiSeq data processing and analysis

Raw reads, generated across six independent MiSeq runs, were processed using PEAR and imported into QIIME2 (Hall and Beiko, 2018) for quality filtering, denoising with DADA2 (Callahan et al., 2016), and amplicon sequence variants (ASV) generation. Taxonomic assignment was performed using a naïve Bayes classifier trained on SILVA SSU database v138 database (Chandel et al., 2022b; Ramakodi, 2022). Diversity metrics were calculated within QIIME2 and visualised in R (v4.3.1) using the Phyloseq package (McMurdie and Holmes, 2013). Core taxa were defined as those present in ≥90% of samples within each species. To estimate culturability, ASVs were BLASTn-matched (≥96% identity) against a 16S database constructed from the isolate genomes. Detailed parameters and full analytical workflows are provided in Supplementary Section 2.

## 3 Results

### 3.1 Overview of sequence processing and ASV recovery

High throughput 16S rRNA gene amplicon sequencing was performed on 864 seedling samples derived from 36 *Medicago* seed accessions across six MiSeq runs. Following QIIME2 processing—including paired-end read merging, denoising, and quality filtering—825 high-quality amplicon libraries were retained. The pipeline also included removal of low-abundance features (frequency < 10), exclusion of features present in fewer than two samples, and taxonomic filtering to eliminate eukaryotic, mitochondrial, and chloroplast-derived sequences. The final dataset comprised 70,258,729 high-quality sequences clustered into 719 ASVs. Of these, 22,295,615 sequences (31.73%) originated from domesticated lucerne and were assigned to 330 ASVs, while 47,963,114 sequences (68.27%) derived from CWRs, corresponding to 389 ASVs. To normalise sequencing depth across samples, rarefaction was applied at 5,061 reads per sample, resulting in 153 ASVs retained across 717 samples. Taxonomy subsequently collapsed to the genus level, yielding 107 unique genera. The relative abundance of all retained genera across both domesticated lucerne and CWR accessions are presented in Supplementary Table 1.

### 3.2 Diversity patterns in the *Medicago* seed microbiome

#### 3.2.1 Alpha diversity

**FIGURE 2:**
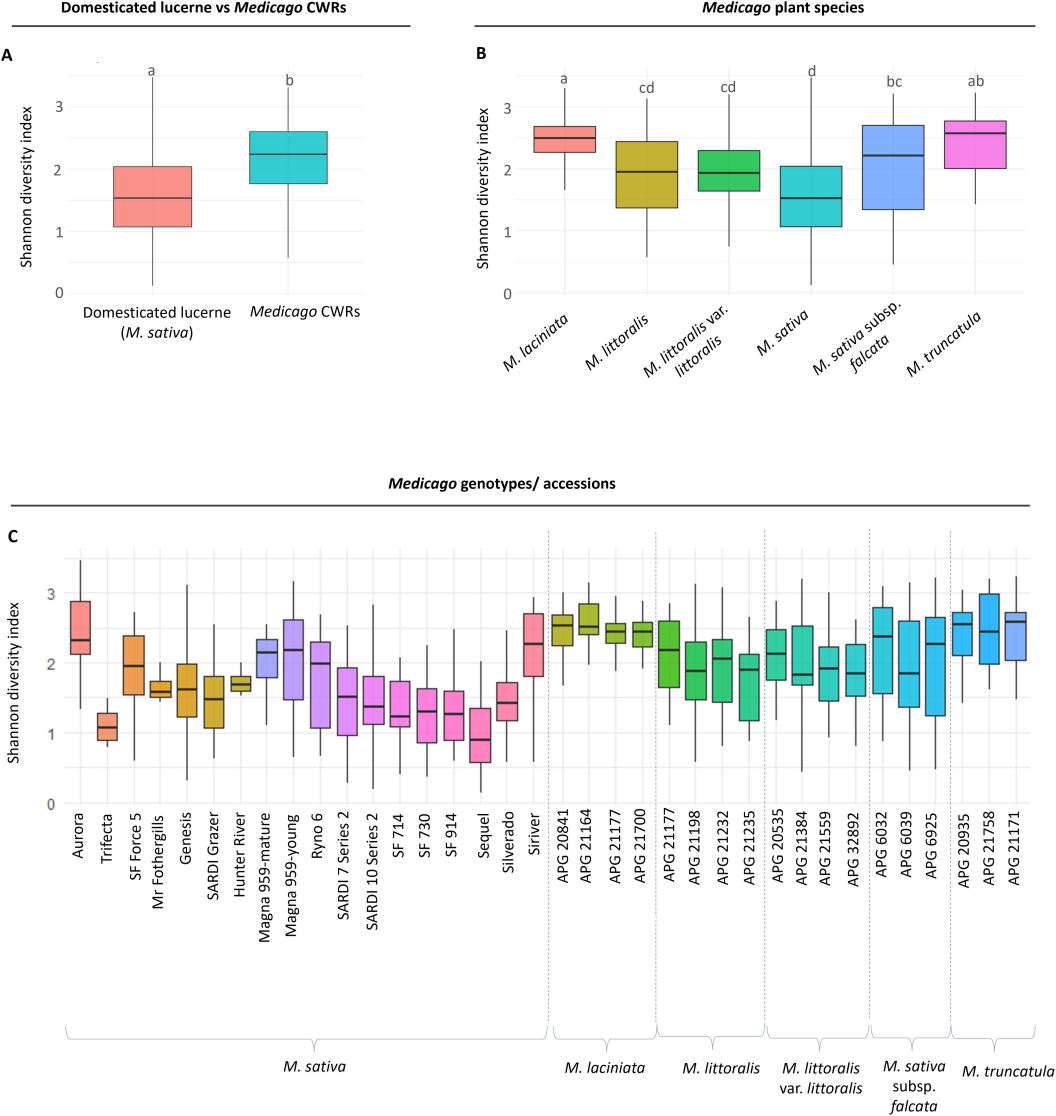
Alpha diversity of seed microbiomes across domesticated lucerne and crop wild relatives (CWRs). Shannon diversity indices were calculated based on amplicon sequence variants (ASV) data. **(A)** Comparison of domesticated lucerne (*Medicago sativa*, *n* = 323) and CWRs (*n* = 394) shows significantly higher diversity in wild accessions. Different letters indicate statistically significant differences (Kruskal–Wallis test, *p* < 0.05 CWRs), **(B)** diversity comparisons across individual *Medicago* species reveal interspecific variation. **(C)** Shannon diversity across accessions within domesticated and wild groups highlights accession-level differences. Each boxplot shows median, interquartile range (IQR), and whiskers extending to 1.5 × the IQR.

Alpha diversity was assessed using the Shannon diversity index to quantify within-sample bacterial richness across all retained samples (*n* = 717). Across the dataset, *Medicago* CWRs exhibited significantly higher bacterial diversity compared to domesticated lucerne, with mean Shannon indices of 2.15 and 1.55 respectively (*p* = 1.16E-29) (Figure 2A and Supplementary Table 2). When grouped by plant species, *M. laciniata* harboured significantly more diverse seed-associated bacterial community than *M*. *littoralis* (*p* = 1.74E-10), *M. littoralis* var. *littoralis* (*p* = 1.53E-10) and *M. sativa* (*p* = 2.86E-29) (Figure 2B and Supplementary Table 3). Within domesticated group, diversity was significantly lower than *M. sativa* subsp. *falcata* (*p* = 1.21E-05) and *M. truncatula* (*p* = 1.82E-16), further highlighting the potential effects of domestication on seed microbiome diversity (Supplementary Table 3). At the accession level, *M. laciniata* APG 21164 displayed the highest alpha diversity (Shannon index = 2.57), while the Sequel accession of domesticated lucerne had the lowest (0.97) (Figure 2C). Within the domesticated group, the Aurora seed accession showed the highest diversity (2.37), suggesting considerable genotype-level variation within domesticated lines (Figure 2C).

#### 3.2.2 Beta diversity

**FIGURE 3:**
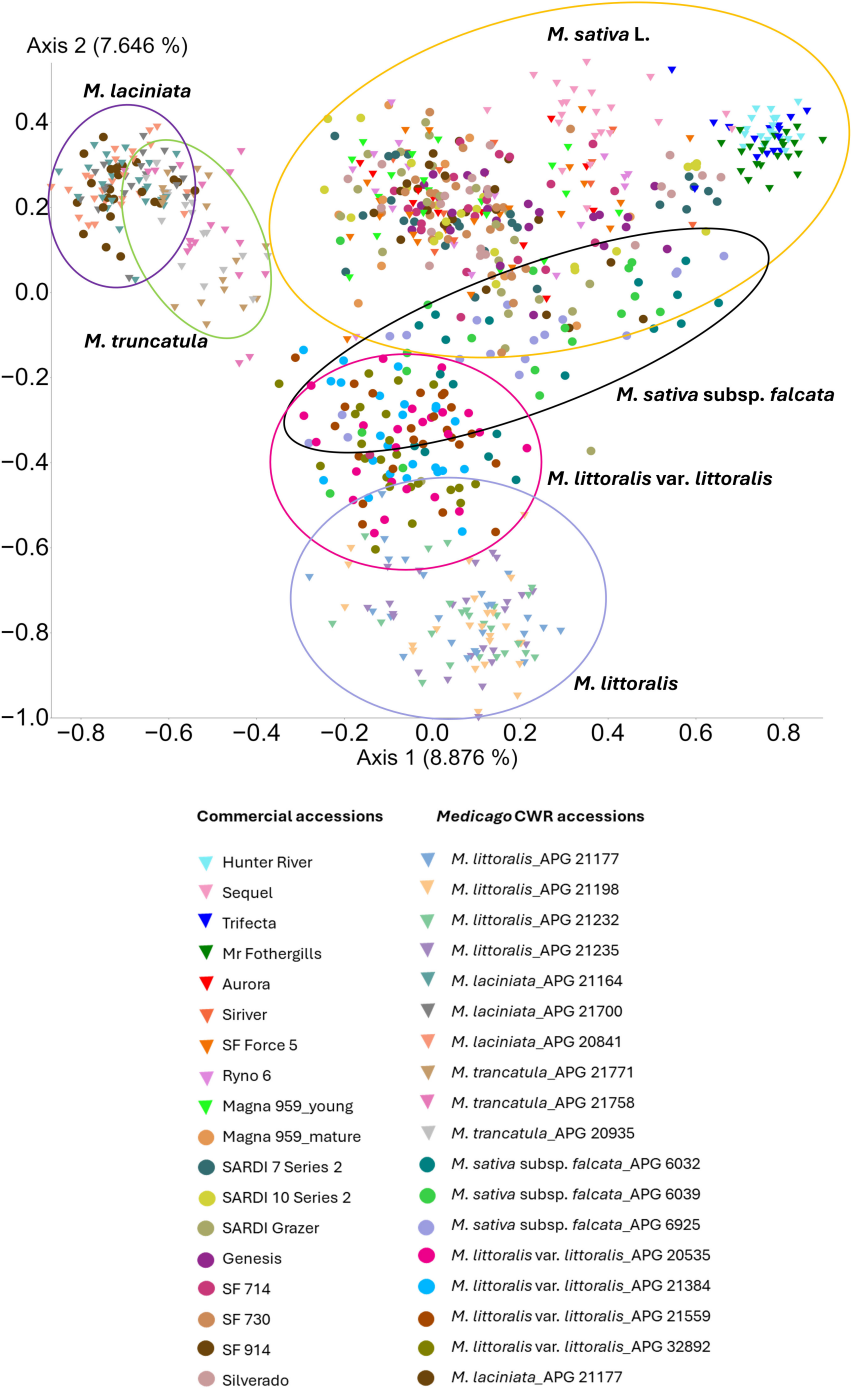
Principal coordinate analysis (PCoA) of seed-associated bacterial communities across 36 *Medicago* accessions. Ordination is based on Jaccard dissimilarity of ASV presence–absence data. Each point represents a single seedling, colour-coded by accession. Variation in community composition is projected along the first two principal coordinate axes. Triangles indicate accessions included in both microbiome profiling and culturable isolate recovery, whereas circles represent accessions analysed only through microbiome profiling.

Beta diversity analysis revealed clear differences in bacterial community composition across *Medicago* species. Principle Coordinates Analysis (PCoA) based on Jaccard dissimilarity showed distinct clustering of samples by plant species, with domesticated lucerne accessions forming a centralised cluster, while *Medicago* CWRs exhibited more dispersed, species-specific groupings (Figure 3). Statistical comparisons using unweighted UniFrac distances confirmed that bacterial community composition differed significantly across host species (PERMANOVA, *p* < 0.05; Supplementary Table 4), consistent with the ordination patterns. In addition to these compositional differences (differences in community centroids), tests for homogeneity of group dispersion (PERMDISP) revealed significant variability in the degree of within-group dispersion across several species pairs. Notable contrasts in dispersion were observed between *M. laciniata* and *M. sativa*, as well as *M. laciniata* and *M. truncatula*; between *M. littoralis* and each of *M. littoralis* var. *littoralis*, *M. sativa*, and *M. truncatula*; between *M. littoralis var. littoralis* and *M. sativa*; between *M. sativa* and both *M. sativa* subsp. *falcata* and *M. truncatula*; and between *M. sativa* subsp. *falcata* and *M. truncatula* (Supplementary Table 4). These results indicate that both shifts in average community composition and differences in variability of community structure contribute to species-level differentiation in the *Medicago* seed microbiome.

### 3.3 Taxonomic analysis of *Medicago* seed microbiome

#### 3.3.1 Overview of taxonomic composition

Taxonomic profiling of seed-associated bacterial communities was performed at the phylum, class, and genus levels to evaluate patterns of community composition across *Medicago* host species and seed accessions. Analyses were stratified by both plant species and genotypes to assess the relative influence of host taxonomy and genotype/cultivar on microbiome structure.

At the phylum level, eight bacterial phyla were detected across all samples, excluding unassigned groups representing less than 0.1% of total sequence reads (Supplementary Table 5). The dominant phyla—Proteobacteria, Firmicutes, and Actinobacteria—were consistently abundant in both domesticated lucerne and *Medicago* CWRs (Supplementary Figure 1 and Supplementary Table 5) and were prevalent across all 36 seed accessions (Supplementary Table 6). Proteobacteria was the most abundant, comprising 46.3–99.9% of the total bacterial community across accessions, followed by Firmicutes (0.005–53.2%) (Supplementary Figure 2).

At the class level, 11 bacterial classes were identified, with Gammaproteobacteria (75.0–77.2%), Bacilli (20.5–21.6%), Actinobacteria (0.76–1.07%), and Alphaproteobacteria (0.44–2.96%) being the most abundant overall (Supplementary Figure 3 and Supplementary Table 7). Notably, Alphaproteobacteria was more enriched in *Medicago* CWRs (mean relative abundance = 2.96%) compared to domesticated lucerne (0.44%), representing a key compositional distinction between wild and domesticated host groups. Across all accessions, Gammaproteobacteria remained the dominant class, with the *M. sativa* cultivar “Sequel” exhibiting the highest relative abundance (99.94%) (Supplementary Figure 4 and Supplementary Table 8).

#### 3.3.2 Taxonomic composition at the genus level

**FIGURE 4:**
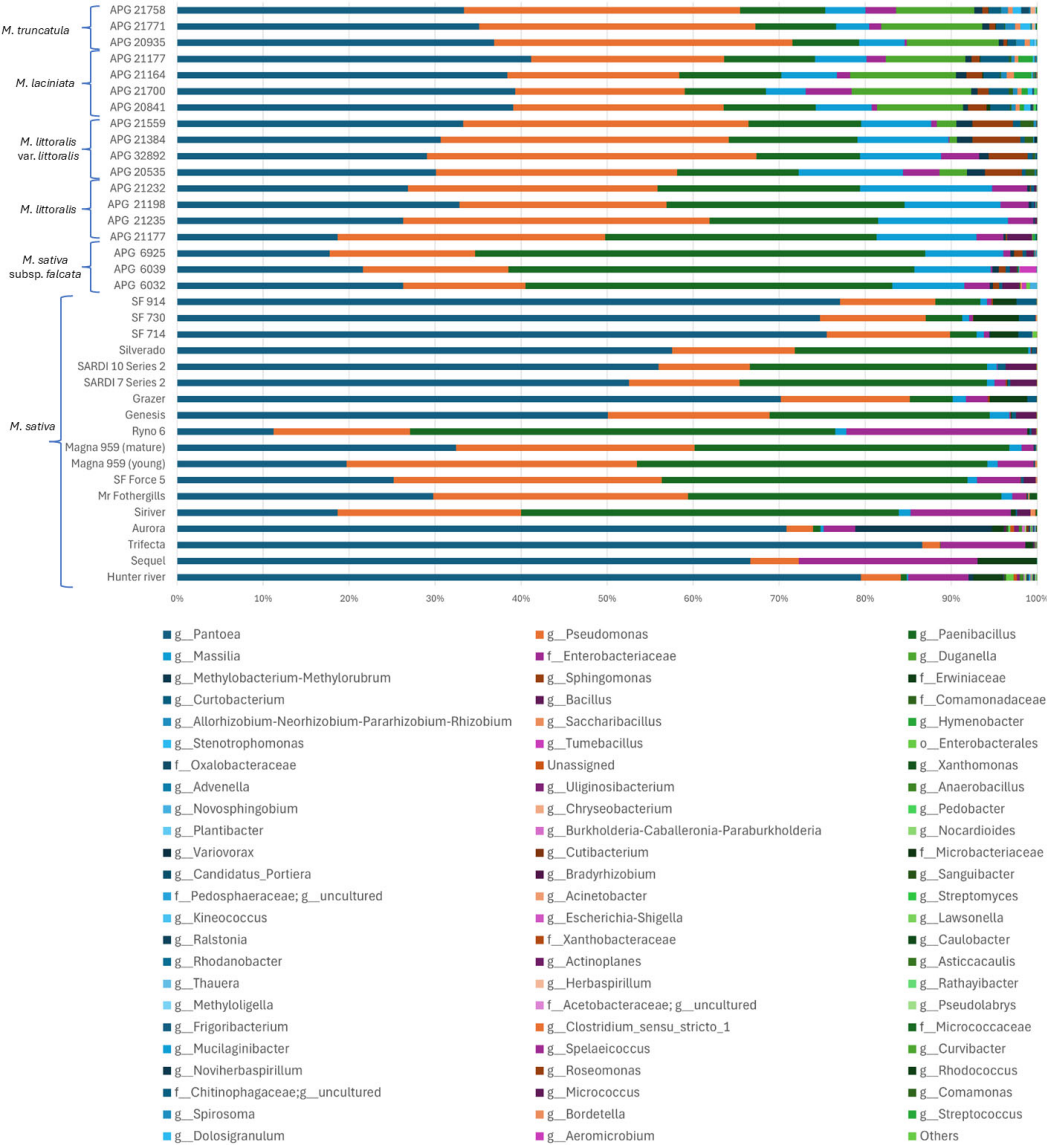
Genus level relative abundance of bacterial communities across *Medicago* seed accessions. Stacked bar plots show the relative abundance of bacterial genera detected by 16S rRNA gene amplicon sequencing across 36 *Medicago* seed accessions, including both domesticated lucerne and CWRs. Each horizontal bar represents a single accession. Genera with relative abundance >0.01% in at least one accession are individually shown; less abundant taxa are grouped under “Others.” Taxonomic assignments are displayed at the genus level or at the lowest available taxonomic rank.

Genus-level profiling of *Medicago* seed microbiome identified 107 bacterial genera across all samples. Of these, 17 genera were detected in domesticated lucerne accessions and 28 in CWRs, each at relative abundance exceeding 0.01%. Genera below this threshold were grouped under “Others” (Figure 4). In domesticated lucerne, the bacterial community was dominated by *Pantoea* (53.64%), *Paenibacillus* (20.63%), *Pseudomonas* (16.59%), along with lower contributions from taxa affiliated with *Enterobacteriaceae* (3.96%) and *Erwiniaceae* (1.80%). In contrast, the most abundant genera across *Medicago* CWR accessions were *Pantoea* (31.12%), *Pseudomonas* (27.25%), *Paenibacillus* (19.80%), *Massilia* (8.93%), and *Duganella* (4.56%) (Supplementary Table 9). Of the 107 identified genera, 27 were shared between domesticated and wild accessions. Twenty genera were unique to CWRs, 13 of which exceeded 0.01% threshold—including *Duganella* (4.56%), *Hymenobacter* (0.32%), and *Tumebacillus* (0.11%). Eighteen genera were exclusive to domesticated lucerne, although only *Xanthomonas* surpassed 0.01% relative abundance (0.07%).

To assess the influence of host genotype/cultivar on bacterial composition, genus-level bacterial profiles were examined across all 36 *Medicago* seed accessions. The three most abundant genera across accessions were *Pantoea* (33.36–86.63%), *Pseudomonas* (2.08–38.32%), and *Paenibacillus* (0.004–52.30%) (Figure 4 and Supplementary Table 1). Notably, *Massilia* was enriched in accessions of *M. littoralis* (11.59–15.36%) and *M. littoralis* var. *littoralis* (9.46–12.10%) but was substantially less abundant in domesticated lucerne (0.00–2.28%). Similarly, *Duganella* was prevalent in *M. laciniata* (9.26–13.87%), *M. truncatula* (9.09–11.72%), and *M. littoralis* var. *littoralis* (0.02–3.21%), but was undetectable in domesticated accessions. The genus *Methylobacterium-Methylorubrum* was particularly enriched in the domesticated cultivar “Mr Fothergills.” Other taxa also exhibited accession-specific enrichment. For example, *M. littoralis* var. *littoralis* showed elevated levels of *Sphingomonas* (4.31–5.60%). In *M. laciniata*, *Hymenobacter* also showed relatively high abundance (0.50–2.05%). *M. truncatula* APG 21771 accession had the highest recorded levels of *Allorhizobium-Neorhizobium-Pararhizobium-Rhizobium* (ANPR-complex) (1.13%) and *Stenotrophomonas* (1.14%). Notably, *M. sativa* subsp. *falcata* was the only species to harbour *Tumebacillus* at >0.01% relative abundance (0.014–1.96%).

To explore whether developmental stage influences microbiome composition, we compared young and mature seeds of the domesticated cultivar “Magna-959.” Both seed stages shared the same dominant taxa, with only minor shifts observed, most notably a reduction in *Bacillus* abundance in mature seeds (Supplementary Figure 5 and Supplementary Table 1). These results suggest compositional stability across seed maturation stages in this cultivar, though broader comparisons across multiple accessions would be required to confirm whether this reflects a general trend.

### 3.4 Core *Medicago* seed microbiome

One of the key aims of this study was to determine whether a conserved core microbiome is shared across the 36 *Medicago* seed accessions. Following the framework proposed by Huse et al. (2012), the core microbiome was defined as bacterial taxa present in more than 90% of samples, irrespective of their relative abundance. This prevalence-based definition allows for the inclusion of taxa that may be functionally important but are not necessarily dominant in abundance. Factors such as host species, genotype, storage conditions, and geographic origin were not used as exclusion criteria for core membership.

#### 3.4.1 Core *Medicago* seed microbiome: species-level perspective

A total of six core bacterial taxa were identified across the *Medicago* seed microbiome, defined by their presence in >90% of samples across all 36 accessions. These taxa represented 5.61% of all taxa detected yet accounted for 3,596,021 sequences—comprising 99.1% of all sequences assigned to the core. This indicates that core membership was largely driven by high-abundance taxa, with the exception of one taxon classified only at the domain level (Bacteria), which contributed <0.1% of core reads. Although unresolved at lower taxonomic levels, this taxon was consistently detected and may represent one or more conserved but poorly characterised bacterial lineages.

**FIGURE 5:**
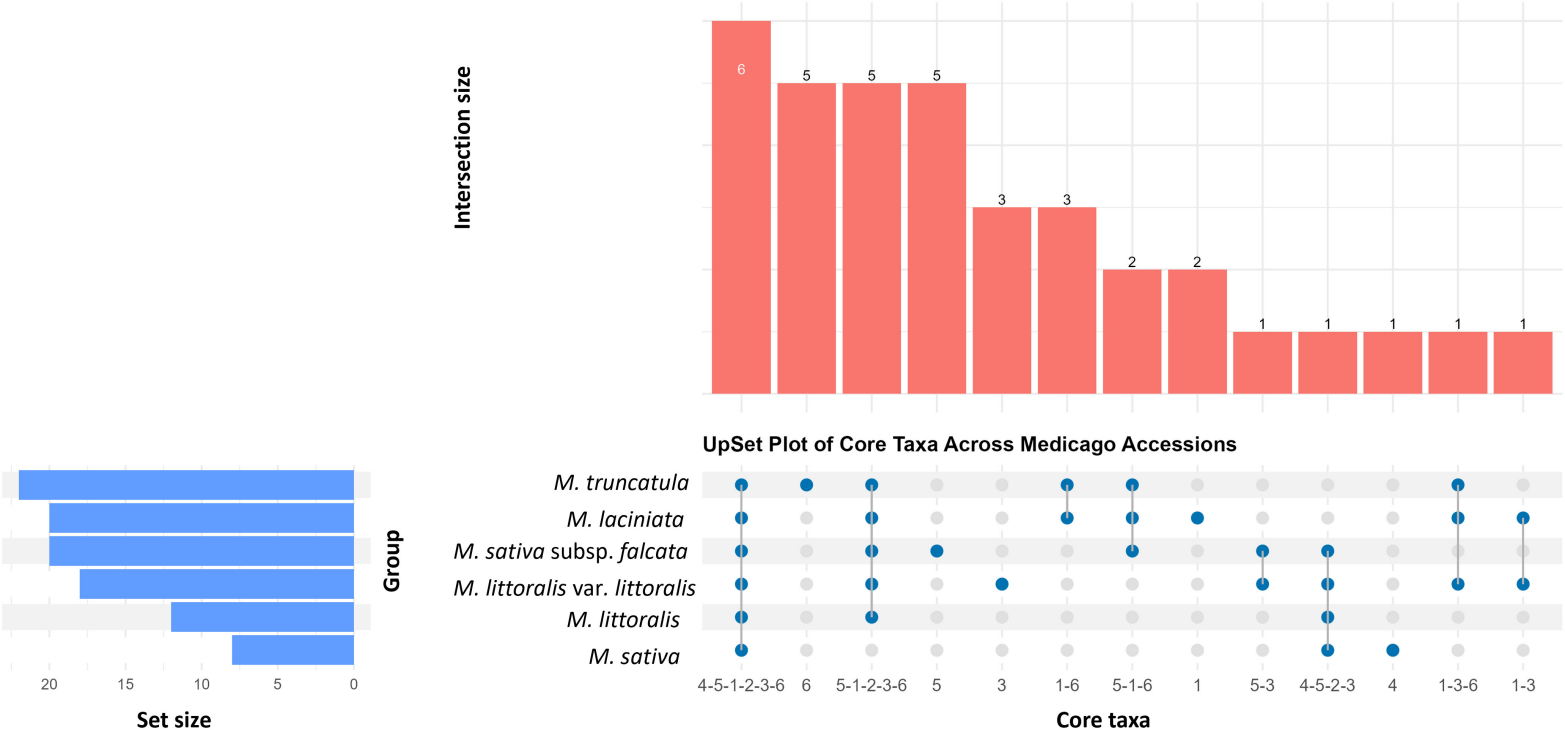
UpSet plot illustrating the distribution of core bacterial taxa (present in >90% of samples) across six *Medicago* species. Vertical bars represent the number of taxa shared among accessions, as indicated by the filled blue dots in the matrix in the bottom panel. The largest intersection (*n* = 6) corresponds to bacterial taxa consistently detected across all *Medicago* species, representing the shared core microbiome. Smaller intersections reflect taxa shared by specific subsets or uniquely associated with individual plant species. Horizontal bars on the left indicate the total number of core taxa identified in each *Medicago* species. This visualisation supports the identification of both conserved and species-specific components of *Medicago* seed core microbiome.

Of the 3,596,021 sequences assigned to core taxa, 3,189,272 (88.69%) were shared among all six *Medicago* species, indicating a predominantly conserved core seed microbiome. These core taxa were taxonomically classified as *Pantoea* (21.87–53.64%), *Pseudomonas* (16.02–33.20%), *Paenibacillus* (9.01–47.37%), Enterobacteriaceae (1.33–3.96%), *Curtobacterium* (0.21–2.46%), and one unclassified taxon assigned only at the domain level (Bacteria) (0.001–0.045%) (Figure 5; Supplementary Table 10).

Species-specific core taxa were also identified. *M. sativa* subsp. *falcata* uniquely harboured core taxa affiliated with Enterobacteriales, *Tumebacillus*, *Novosphingobium*, *Azospirillum*, *Dermococcus*, collectively accounting for 1.28% of its core sequences. *M. laciniata* unique core members included *Frigoribacterium* and Oxalobacteraceae (0.14%), while *M. littoralis* var. *littoralis* contributed *Variovorax*, *Bradyrhizobium*, and *Kineococcus* (0.12%). *M. truncatula* harboured a distinct subset of Actinobacteria—including Microbacteriaceae, *Microbacterium*, *Sanguibacter*, *Chryseobacterium* and *Plantibacter*—accounting for 0.54% of its core reads. *M. sativa* contained Erwiniacea (1.80%) as its only unique core taxon. No unique core taxa were detected in *M. littoralis*, suggesting its core microbiome may be broadly nested within that of other species.

#### 3.4.2 Core seed microbiome of domesticated lucerne versus *Medicago* CWRs

**FIGURE 6:**
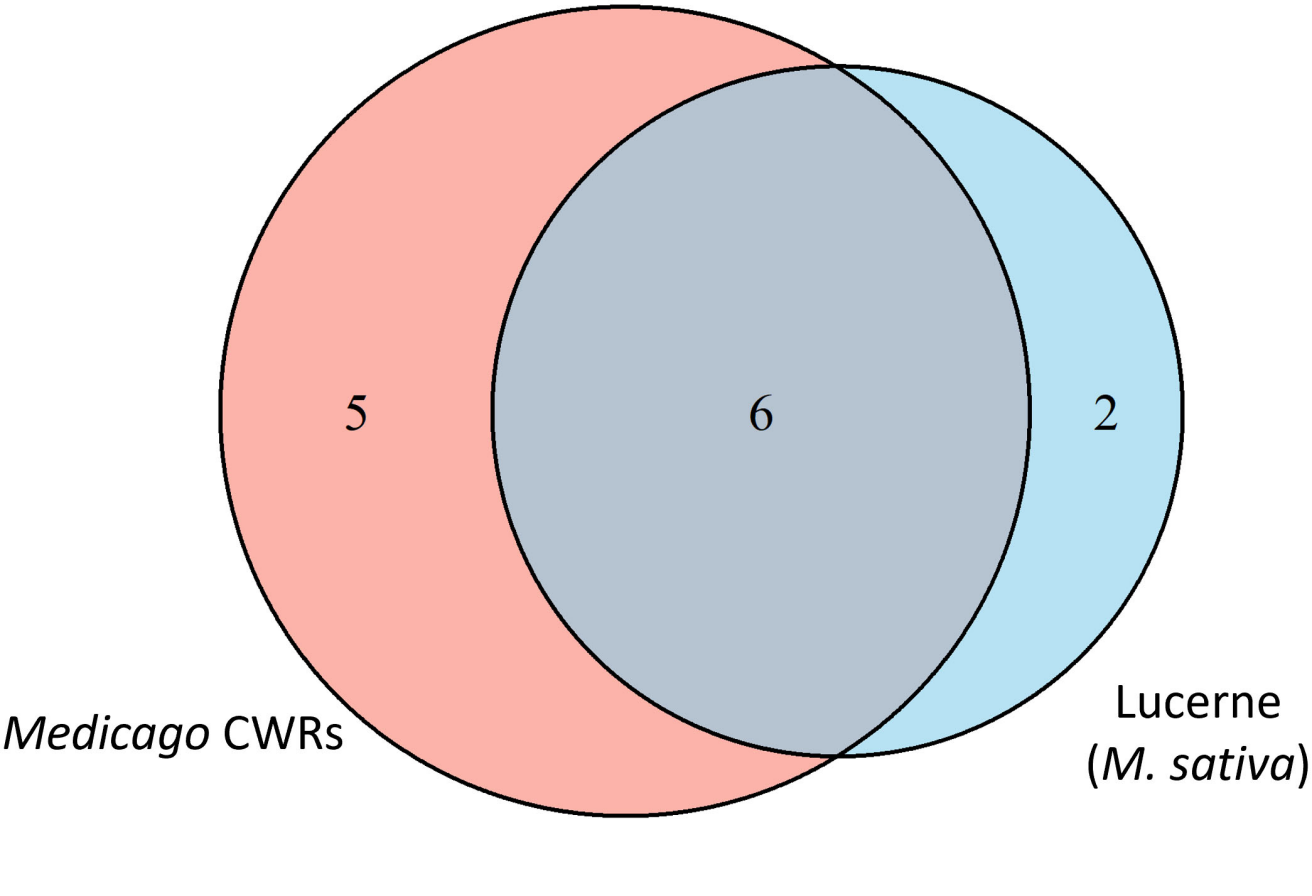
Venn diagram representing the unique and shared bacterial core taxa associated with domesticated lucerne and *Medicago* CWR seeds. The numbers in the intersections are the shared core taxa and the remaining numbers are unique core taxa of both domesticated lucerne and CWRs.

To compare the core community structure between domesticated lucerne and *Medicago* CWRs, core taxa shared across host groups were analysed. Of the 3,189,272 sequences comprising the shared core microbiome (defined by presence in > 90% of samples), 1,562,585 sequences (49.0%) originated from domesticated lucerne and 1,626,687 sequences (51.0%) from CWR accessions. Taxonomic overlap analysis revealed that 75% of the domesticated lucerne core was also present in CWRs, whereas only 54.6% of the CWR core overlapped with that of domesticated lucerne. This asymmetry suggests a broader core diversity in wild relatives and supports the hypothesis that domestication may have filtered out part of the ancestral seed microbiota. Unique core members in domesticated lucerne included *Bacillus* and Erwiniaceae, which were not detected in the core of any CWR species. In contrast, CWR-specific core taxa included *Massilia*, Comamonadaceae, *Sphingomonas*, *Methylobacterium-Methylorubrum* and ANPR-complex. These taxa were consistently detected at >90% prevalence within wild accessions but absent or inconsistently detected in domesticated lucerne (Figure 6).

At the class level, the core microbiome was dominated by Gammaproteobacteria (76.02%), followed by Bacilli (22.96%), Actinobacteria (1.00%). A single core taxon classified only at the domain level (Bacteria) accounted for a minor fraction (0.03%) of total core reads. This taxon lacked assignable lower-level taxonomy and may represent one or more uncharacterised bacterial lineages that were consistently detected but unresolved using current reference databases. At the genus level, the most dominant and widely shared core taxa across both groups were *Pantoea* (19.46–27.50%), *Pseudomonas* (8.50–17.04%), and *Paenibacillus* (10.58–12.38%) (Supplementary Table 11). These taxa likely represent conserved, ecologically adapted, and potentially functionally significant members of *Medicago* seed microbiome.

### 3.5 Microbial isolation and genomic characterisation

#### 3.5.1 Culture-based recovery and taxonomic identification

A total of 530 bacterial isolates were recovered from 19 *Medicago* seed accessions, including nine domesticated lucerne accessions (*n* = 213; 40%) and ten *Medicago* CWR accessions (*n* = 317; 60%). Of these, taxonomic identification was performed on 315 isolates, using either Sanger sequencing of the 16S rRNA gene (*n* = 305; NCBI GenBank BioProject PRJNA1180717) or whole-genome sequencing (*n* = 10; NCBI GenBank BioProject PRJNA1210666). Most identifications were resolved at the genus level, with only a few isolates (*n* = 2) assigned at higher taxonomic ranks due to lower sequence similarity. In total, 37 bacterial genera were represented among the cultured isolates. The culturable community was dominated by *Pantoea* (*n* = 119), followed by *Pseudomonas* (*n* = 29), *Curtobacterium* (*n* = 20), *Erwinia* (*n* = 21), *Paenibacillus* (*n* = 19), *Duffyella* (*n* = 18) and *Bacillus* (*n* = 14), reflecting a mixture of conserved and host-specific taxa within *Medicago* seed microbiome.

#### 3.5.2 Whole-genome sequencing and comparative genomics

To explore the phylogenetic diversity and potential novelty within the culturable seed community, 34 representative bacterial isolates were selected for long-read whole-genome sequencing. Selection criteria prioritised taxa detected in at least one *Medicago* host species, with an emphasis on ecological relevance and representation within the seed microbiome. These selected strains included members of *Pantoea*, *Paenibacillus*, *Pseudomonas*, *Massilia*, Enterobacteriaceae and *Duganella* isolates.

To evaluate taxonomic placement and potential novelty, ANI analysis was conducted by comparing the sequenced genomes to their closest NCBI reference genomes (Supplementary Table 12). Fourteen isolates exhibited ANI values ranging from 96.46 to 99.85%, exceeding the 95% species delineation threshold (Jain et al., 2018). For example, isolate Lu_LA164_018 shared 94.58% ANI with *P. orientalis* (GenBank: GCF_003852045.1), suggesting it may represent a closely related, yet potentially novel, species. An additional, 14 isolates displayed ANI values between 80.43–91.76% relative to their closest references—above the genus-level threshold (>75%) (Wang et al., 2016), but below the species-level cut-off. These genomes likely represent divergent or previously uncharacterised species within the *Medicago* seed microbiome.

To assess intra-species genomic variation, pairwise ANI comparisons were performed among isolates assigned to the same species (Supplementary Table 13). Notably, two *P. fluorescens* strains isolated from the same lucerne cultivar (SF Force 5) Lu_F5_006 and Lu_F5_029 exhibited a 99.9945% ANI, indicating they are closely related but genomically distinct. Similar strain-level divergence was observed in *Duffyella gerundensis* (Lu_R6_023 vs Lu_F5_028; 98.85% ANI), and *Paenibacillus nuruki* (Lu_LT198-042 vs Lu_TR771_007; 98.50% ANI), despite being isolated from different host species. Six isolates of *P. alli* consistently exceeded 99% ANI. Among these, pairs such as Lu_TR758_015 and Lu_TR758_007 (from *M. truncatula*), and Lu_LT198_018 and Lu_LT198_002 (from *M. littoralis*), shared 99.99% similarity, supporting the presence of distinct, coexisting strains within individual host taxa.

### 3.6 Assessing the culturability of the abundant *Medicago* seed-associated taxa

**FIGURE 7:**
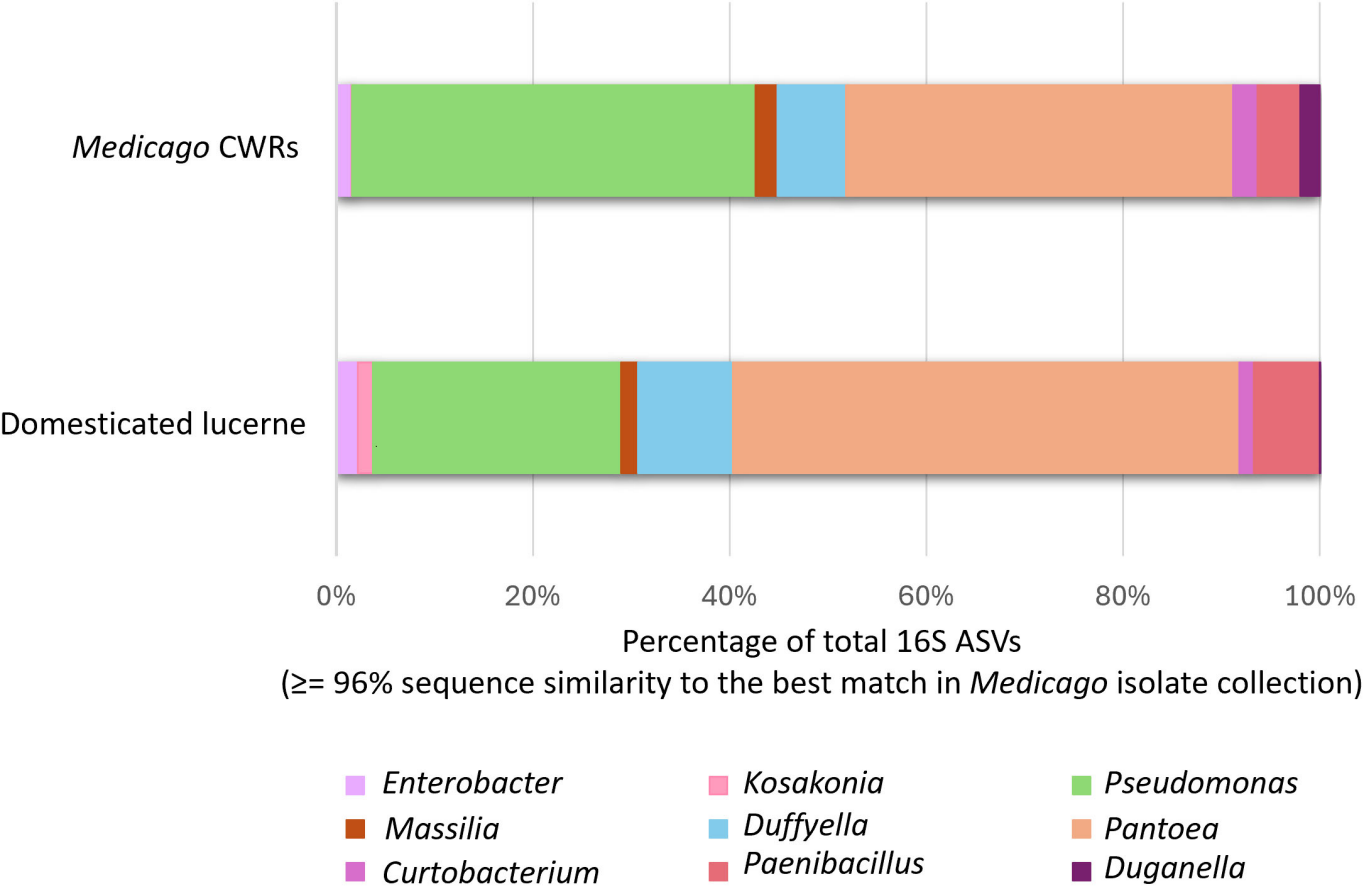
Culturability of abundant bacterial taxa in the *Medicago* seed microbiome. Bar plots show the proportion of total 16S rRNA ASVs from domesticated lucerne and CWRs matching cultured isolates at ≥96% identity. Only ASVs affiliated with abundant cultured genera are shown. Taxa were classified as culturable based on sequence alignment to an in-house genome database constructed from 34 representative isolates.

To assess the culturability of abundant members of the *Medicago* seed microbiome, an in-house BLAST database was constructed using whole-genome assemblies of 34 representative bacterial isolates. These isolates were selected based on their occurrence across multiple host accessions and affiliation with high-abundance genera identified through 16S rRNA gene profiling. ASVs generated via QIIME2 were aligned to the isolate genome database using BLASTn. ASVs with ≥96% sequence identity to a reference genome were classified as culturable under the current experimental conditions, while those falling below this threshold were designated as non-culturable. The ≥96% threshold was chosen as a conservative criterion to minimise false negatives when aligning short-read ASVs to isolate genomes, and was intended for estimating culturability rather than for genus- or species-level delineation. Using this approach, 55.71% of abundant ASVs from domesticated lucerne and 63.39% from *Medicago* CWRs matched cultured representatives at ≥96% identity. The most frequently cultured ASVs were affiliated with the genera *Pantoea*, *Pseudomonas* and *Duffyella* (Figure 7), indicating that several dominant community members were successfully captured through cultivation.

Nevertheless, a substantial proportion of abundant ASVs—44.29% in domesticated lucerne and 36.61% in CWRs—remained uncultured under the applied isolation protocols. These should be interpreted as unculturable under the specific media and conditions employed, recognizing that additional taxa may be recovered using alternative approaches. As only 34 isolates were genome-sequenced, the analysis provides an indicative estimate of culturability for the abundant fraction of the seed microbiome rather than an exhaustive representation of community diversity. This strategy of sequencing a subset is consistent with previous seed microbiome study by Chandel et al. (2022b), which similarly used a fraction of the cultured isolate collection for genome-resolved culturability analyses.

## 4 Discussion

### 4.1 Mapping the microbial blueprint: profiling the *Medicago* seed microbiome

This study demonstrated that *Medicago* seeds predominantly hosted bacterial taxa from the classes *Gammaproteobacteria*, *Bacilli*, *Actinobacteria* and *Alphaproteobacteria* (Supplementary Figure 3). This taxonomic structure aligns with seed microbiome profiles reported in other agronomically important species, including perennial ryegrass (*Lolium perenne*) (Tannenbaum et al., 2020), Soybean (*Glycine max*) (Chandel et al., 2022a; Chandel et al., 2022b), rice (*Oryza sativa*) (Nakaew and Sungthong, 2018; Eyre et al., 2019; Kim et al., 2020), barley (*Hordeum vulgare*) (Bziuk et al., 2021) and bread wheat (*Triticum aestivum*) (Kuźniar et al., 2020; Hone et al., 2021). Unlike studies focused solely on cultivated lines, the inclusion of both domesticated lucerne and diverse *Medicago* CWRs offers a rare comparative framework to examine microbiome conservation and divergence in the context of domestication.

The CWRs exhibited a higher relative abundance of Alphaproteobacteria, including genera such as *Bradyrhizobium* and members of the ANPR-complex compared to domesticated lucerne (Supplementary Tables 7, 9). Two *Rhizobium*-affiliated isolates (Lu_LT198_001 and Lu_TR935_W004) were also recovered from CWR accessions (Supplementary Table 14), suggesting that vertical transmission of mutualistic bacteria may be retained within wild *Medicago* lineages—consistent with previous reports in *M. truncatula* (Brown et al., 2020; Burns et al., 2021). The dominant genera in this study, *Pantoea*, *Paenibacillus*, and *Pseudomonas* were also detected in culturable seed microbiomes of domesticated lucerne from Argentina (López et al., 2018), where seed-associated bacteria were shown to persist under favourable storage conditions. Similar taxonomic patterns observed in the seed microbiome of Styrian oil pumpkin (*Cucurbita pepo*) (Adam et al., 2018), further support the concept of conserved microbial assemblages across diverse plant species. Notably, several lucerne cultivars obtained as certified organic seed lots (Hunter River, Sequel, Eden, Aurora, and Siriver) exhibited an increased prevalence of Enterobacteriaceae (Supplementary Table 1). As all 36 accessions were cultivated under the same controlled conditions in this study, this distinction reflects seed source rather than experimental treatment. This pattern is consistent with findings from organic raspberry cultivation systems (Sangiorgio et al., 2021), and studies in *M. truncatula* (Kêpczyńska and Karczyński, 2019), where members of Enterobacteriaceae with recognised PGP traits were also enriched.

In one domesticated cultivar (“Magna-959”), young and mature seeds displayed broadly similar bacterial assemblages, with only a decline in *Bacillus* in mature seeds, distinguishing the developmental stages. While this preliminary observation hints at a degree of microbiome stability during maturation, this finding is based on a single accession and should be interpreted cautiously. Broader comparative analyses will be necessary to establish whether seed maturation consistently acts as a selective filter favouring microbial taxa adapted to desiccation and dormancy (Leprince et al., 2017; Chesneau et al., 2020).

### 4.2 Domestication’s footprint: probing the lucerne seed microbiome

Our findings support the hypothesis that domestication has significantly altered the composition and diversity of *Medicago* seed-associated microbiomes. Seeds of CWRs consistently harboured more diverse and compositionally distinct bacterial communities than their domesticated counterparts (Figure 2A), reflecting patterns reported in *Glycine* species (Chandel et al., 2022b) and alpine cropping systems (Wassermann et al., 2019). Among *Medicago* accessions, *M. laciniata* exhibited the greatest bacterial richness, whereas domesticated lucerne displayed the lowest (Figure 2B). This reduction in diversity likely reflects the genetic bottlenecks imposed during domestication and selective breeding (Buckler et al., 2001). Nuclear DNA polymorphism analyses indicate an estimated 30% loss of genetic diversity in domesticated lucerne relative to its wild progenitors, despite its autotetraploid nature (Prosperi et al., 2014). Similar declines have been observed in rice (Zhu et al., 2012), soybean (Hyten et al., 2006), and maize (Wright et al., 2005), where selective sweeps targeting agronomic traits have also reduced diversity in adjacent genomic regions (Clark et al., 2004; Palaisa et al., 2004; Sweeney and McCouch, 2007). These genomic constraints may limit not only allelic variation but also the plant’s capacity to recruit or retain beneficial microbial partners. It is also important to note that the CWR accessions included multiple *Medicago* species (*M. sativa* subsp. *falcata*, *M. littoralis*, *M. truncatula*, and *M. laciniata*), whereas the domesticated group consisted only of *M. sativa*. This broader taxonomic scope may contribute to the higher richness observed in CWRs. However, our PERMANOVA results revealed significant compositional differences not only between domesticated *M. sativa* and other wild species, but also between domesticated *M. sativa* and its wild progenitor *M. sativa* subsp. *falcata* (*p* = 0.01). Together with significant PERMDISP results (Supplementary Table 4), which indicate greater variability among CWRs and a more uniform community structure in domesticated *M. sativa*, these findings suggest that the diversity contrast reflects both species-level effects and domestication-related bottlenecks.

By contrast, seed microbiomes of CWRs appear more ecologically complex, which may reflect long-term ecological associations with host plants in relatively undisturbed environments. Such associations have been proposed to support the formation of integrated, and potentially functionally diverse microbial consortia (Igwe and Vannette, 2018; Pérez-Jaramillo et al., 2018; Abdullaeva et al., 2021). In wild barley, for example, co-occurrence network analyses have revealed strong host-microbe specificity, indicative of selective microbial assembly (Rahman et al., 2018). In domesticated lucerne, frequent genomic reconfiguration through selective and speed breeding may disrupt these ecological filters, potentially weakening the stability and functional breadth of seed microbiomes (Cordovez et al., 2019). In addition to taxonomic loss, domestication may contribute to functional simplification. Low-abundance or rare taxa, which are frequently lost during domestication, have been shown in other systems to carry specialised traits such as N fixation, antimicrobial compound synthesis, and abiotic stress resilience. Although present at low relative abundance, these taxa can contribute disproportionately to ecosystem function (Tkacz and Poole, 2015; Toju et al., 2018). Their depletion in *Medicago* could therefore reduce functional redundancy and limit the adaptive potential of the plant holobiont, a possibility that warrants further testing.

Domestication-related shifts in microbiota have been documented in soybean (Chandel et al., 2022b), rice (Kim et al., 2020), cereal grasses (Abdullaeva et al., 2021), and wheat (Abdullaeva et al., 2022), and are frequently intensified by modern agricultural practices (Banerjee et al., 2019). Environmental variables such as soil chemistry, pH, fertilisation practices, and organic matter inputs can also influence microbial assembly and function (Fierer and Jackson, 2006; Lauber et al., 2009; Johnston-Monje and Raizada, 2011; Bulgarelli et al., 2015; Kim et al., 2020). While host genotype emerged as the dominant driver in our dataset, environmental filtering during cultivation and seed production likely contributed to shaping microbial community composition.

Moreover, traits commonly targeted during crop improvement such as seed size, seed coat traits, and flowering phenology may inadvertently select for specific microbial assemblages, narrowing microbial diversity across breeding cycles. Microbial filtering that begins at the seed stage may propagate downstream, influencing microbiome composition in roots, rhizosphere, and phyllosphere, with consequences for plant development, immune interactions, and stress adaptation, as suggested in other systems (Nelson, 2018; Trivedi et al., 2020; Abdullaeva et al., 2021).

We also identified low-abundance ( < 0.01%) microbial taxa unique to Australian domesticated lucerne cultivars (Supplementary Table 1). These included genera with plant-beneficial functions such as *Asticcacaulis* (Okazaki et al., 2021), *Mucilaginibacter* (Madhaiyan et al., 2010), *Herbaspirillum* (Estrada et al., 2013) and *Flavobacterium* (Soltani et al., 2010). *Actinoplanes* (El-Tarabily, 2003) have also been associated with biocontrol activity. Interestingly, human-associated taxa such as *Staphylococcus* (Parlet et al., 2019) and *Corynebacterium* (Byrd et al., 2018) were also detected. While their presence may reflect inter-kingdom microbial exchange during seed handling, they have also been reported as persistent, low-abundance constituents of core plant microbiomes (Campisano et al., 2014; Kuźniar et al., 2020), suggesting anthropogenic but non-transient colonisation. These findings raise the possibility that human intervention during seed production and handling may have inadvertently shaped seed microbiomes in domesticated species.

The distinct microbial signatures identified in CWRs highlight their value not only as reservoirs of genetic diversity, but also as sources of ecologically important microbial taxa potentially lost through domestication. These findings highlight the seed as a critical, underexplored entry point in microbiome assembly. Integrating microbiome profiling into breeding pipelines may facilitate the reintroduction of beneficial microbes into elite cultivars, either through targeted genotype selection or seed-applied synthetic communities (SynComs) designed to restore microbial function and improve resilience (Arnault et al., 2024; Luo et al., 2024). In this context, the seed microbiome represents a largely untapped resource for microbiome-informed crop improvement.

### 4.3 Untangling the complexity: identifying key drivers shaping the *Medicago* seed microbiome

The assembly of seed-associated microbiomes is shaped by interacting factors, including host genotype, ecological history, and environmental conditions. While earlier studies have emphasised the role of plant species and geographic origin (Rochefort et al., 2019; Chartrel et al., 2021; Moreira et al., 2021), our findings indicate that host genotype—both at species and cultivar level—emerged as the dominant determinant of bacterial community composition in *Medicago* seeds. This pattern aligns with prior observations in wheat (Kuźniar et al., 2020) and is reinforced by our PCoA results, which revealed distinct clustering of microbiota by *Medicago* species (Figure 3). The close overlap between *M. sativa* and *M. sativa* subsp. *falcata* likely reflects their phylogenetic proximity (Chen et al., 2021). Notably, genotype-driven differences persisted despite uniform cultivation and storage conditions, underscoring the robustness of host genetic influence.

Within domesticated lucerne, cultivars exhibited distinct microbial assemblages, including differences in dominant taxa. For instance, Erwiniaceae were enriched in cultivar “Sequel,” while *Methylobacterium-Methylorubrum* dominated cultivar “Mr. Fothergills” (Figure 3). Even cultivars sourced from the same commercial supplier (Barenbrug Australia Pty Ltd.) displayed divergent profiles. For example, *Paenibacillus* abundance was lower in “SARDI Grazer” than in “SARDI 7 Series 2,” and “SARDI 10 Series 2.” These findings are consistent with previous work in *Arabidopsis*, where genotype-specific responses to *P. fluorescens* influenced plant health (Haney et al., 2015). Species-specific microbial specificity was also evident, with taxa such as *Hymenobacter* and ANPR complex preferentially associated with *M. laciniata* and *M. truncatula*, respectively. Such patterns suggest that plants selectively recruit microbial partners based on genetically encoded traits, potentially shaping microbial inheritance and influencing early plant-microbe interactions.

Although secondary to genotype, cultivation form (wild vs. domesticated) also contributed to microbial variation. CWRs were enriched in beneficial taxa such as *Massilia*, *Duganella*, *Advenella*, and *Hymenobacter*, many of which possess known PGP and stress-mitigating functions (Hrynkiewicz et al., 2010; Aranda et al., 2011; Bonanomi et al., 2018; Raths et al., 2020; Kuzmina et al., 2022; Yarte et al., 2023). In contrast, domesticated accessions showed higher relative abundance of Enterobacteriaceae and Erwiniaceae. Bacterial communities in CWRs exhibited greater dispersion (Supplementary Table 4), likely reflecting increased ecological and genetic diversity, consistent with findings in soybean (Chandel et al., 2022b). This supports the hypothesis that domestication not only narrows plant genetic diversity but also constrains microbial variability.

The strong genotype effect observed here likely stems from host traits that modulate microbial recruitment during seed development and maturation (Bulgarelli et al., 2015; Nelson, 2018). These traits may act as selective filters, enriching microbes capable of persisting through seed desiccation and supporting early-stage growth (Shade et al., 2017; Abdelfattah et al., 2023). The consistent recovery of species- and cultivar-specific taxa, even under standardised conditions, raises questions about the relative contributions of vertical transmission via reproductive tissues and horizontal acquisition from the environment (Shade et al., 2017; Zhang et al., 2022). Although environmental uptake during seed production cannot be excluded, the persistence of specific taxa in genetically related accessions suggest that vertical transmission could contribute alongside environmental acquisition.

Collectively, these findings establish host genotype as the primary architect of seed microbiome structure in *Medicago*, with cultivation forms exerting a secondary but discernible influence. The identification of genotype-specific microbial signatures with plant-beneficial traits suggests opportunities to integrate microbiome selection into breeding pipelines. By understanding how host genetics govern microbial recruitment, it may be possible to design SynComs or breeding strategies that harness microbiome function in cultivated systems—restoring ecological complexity and potentially supporting crop performance.

### 4.4 Validating the culturability of *Medicago* seed microbiome

Culturing seed-associated bacteria expands the resources available for exploring their ecological roles and potential applications. Our study showed that CWRs harboured greater culturable diversity than domesticated lines, a trend that may reflect broader ecological heterogeneity and reduced anthropogenic filtering. These findings reinforce the value of CWRs as microbial reservoirs and are consistent with reports indicating that many ecologically important taxa remain uncultured under standard conditions (Creevey et al., 2014), underscoring the need for improved culturing methods.

Among the most dominant cultured taxa were *Pantoea*, *Erwinia*, *Pseudomonas*, *Paenibacillus*, and *Bacillus*. Several of these, *Pantoea*, *Pseudomonas*, and *Paenibacillus* were also identified as core members of the *Medicago* seed microbiome (Supplementary Table 14). While known to exhibit PGP traits, some strains are pathogenic (Morris et al., 2007; Sebaihia et al., 2010; Walterson and Stavrinides, 2015; Grady et al., 2016; Hernández et al., 2023), highlighting their dual potential and the importance of validating ecological roles at strain-level prior to application. In addition to the dominant genera, several rare or low-abundance taxa, also referred to as satellite taxa and defined as those represented by a single isolate (Rabinowitz, 1981; Hanski, 1982; Dawson et al., 2017) were recovered, including *Burkholderia, Massilia, Novosphingobium, Lysinimonas, and Kocuria*, (see Supplementary Table 14 for rare taxa). Similar taxa have been identified in rice and wheat seed microbiomes (Costa et al., 2014). Although low in abundance, such taxa have been shown in other systems to influence community dynamics by suppressing opportunistic invaders and maintaining stability (Mallon et al., 2015).

To evaluate culturability, we mapped 16S rRNA ASVs to the genomes of cultured isolates, indicating that a substantial portion of the abundant fraction of the seed microbiome in both *Medicago* CWRs and domesticated lucerne could be recovered under the current conditions. This approach provides an estimate of overlap rather than a definitive measure of ecological representativeness. Comparable culturability rates have been reported for ryegrass seed microbiome using R2A medium (Tannenbaum et al., 2020). However, recovery remains influenced by culturing strategies, including media composition, solidifying agents, and culturing protocols. For instance, substituting agar with phytagel in Luria-Bertani (LB) medium has been shown to improve isolation efficiency by altering sugar composition and reducing inhibitory effects (Kato et al., 2020; Wang et al., 2020; Youseif et al., 2021). Additional constraints include nutrient depletion by fast-growing bacteria, high agar concentrations, autoclaving-induced inhibitory compounds, and the accumulation of metabolic by-products (Acuña et al., 2020; Bonnet et al., 2020). Plant-based media, such as lucerne or clover-derived “teabag” cultures have also been shown to enhance the recovery of fastidious taxa (Sarhan et al., 2016; Hegazi et al., 2017), and may improve recovery of functionally important microbes that are otherwise difficult to culture.

### 4.5 The core *Medicago* seed microbiome: stability, functional potential, and relevance for crop resilience

Despite variation in bacterial composition across the six *Medicago* species, a conserved subset of taxa, present in over 90% of samples (See section “3.4 Core *Medicago* seed microbiome**”**), was consistently detected. This core seed microbiome holds agricultural relevance as a foundation for microbial inoculants, including biofertilisers and biocontrol agents (Eyre et al., 2019). These core taxa likely represent conserved microbial partners that persist across *Medicago* genotypes and confer physiological benefits to their hosts. Similar core microbiomes have been reported in soybean (Chandel et al., 2022b), maize (Johnston-Monje and Raizada, 2011) and cereals (Abdullaeva et al., 2021), often independent of geography or domestication status.

The *Medicago* seed microbiome exhibited a relatively small and taxonomically concentrated core, in contrast to crops such as soybean and oilseed rape (*Brassica napus* L.), which harbour core microbiomes comprising 28 and 59 bacterial genera, respectively (Rybakova et al., 2017; Chandel et al., 2022b). *Medicago* core taxa were restricted to a few dominant genera shared across accessions. This streamlined structure reflects findings in alpine seeds, where core taxa constituted only 0.09% of total operational taxonomic units (OTUs), underscoring the strong influence of host genotype on microbiome stability (Wassermann et al., 2019). Similarly, radish (*Raphanus sativus*) seeds hosted individual-specific microbiomes, yet three OTUs accounted for over 70% of total reads per plant, indicating the dominance of conserved seed-adapted bacteria (Rezki et al., 2018). The persistence of these core taxa across *Medicago* species may reflect genotype-driven filtering during seed development, with possible contributions from co-selection process reported in other systems. Traits such as seed coat chemistry, exudate composition, and host immune signalling likely shape microbial recruitment, while vertical transmission across generations may further stabilise these associations (Barret et al., 2015; Mitter et al., 2017). Comparable host–microbe specificity has been reported in seed microbiomes of tobacco (*Nicotiana tabacum*) (Chen et al., 2020) and in rhizobiomes of sugar beet (*Beta vulgaris*) (Zachow et al., 2014), lettuce (*Lactuca sativa*) (Cardinale et al., 2015) and common beans (*Phaseolus vulgaris*) (Mendes et al., 2017), suggesting that such tightly conserved associations may be a broader feature of plant microbiomes.

**FIGURE 8:**
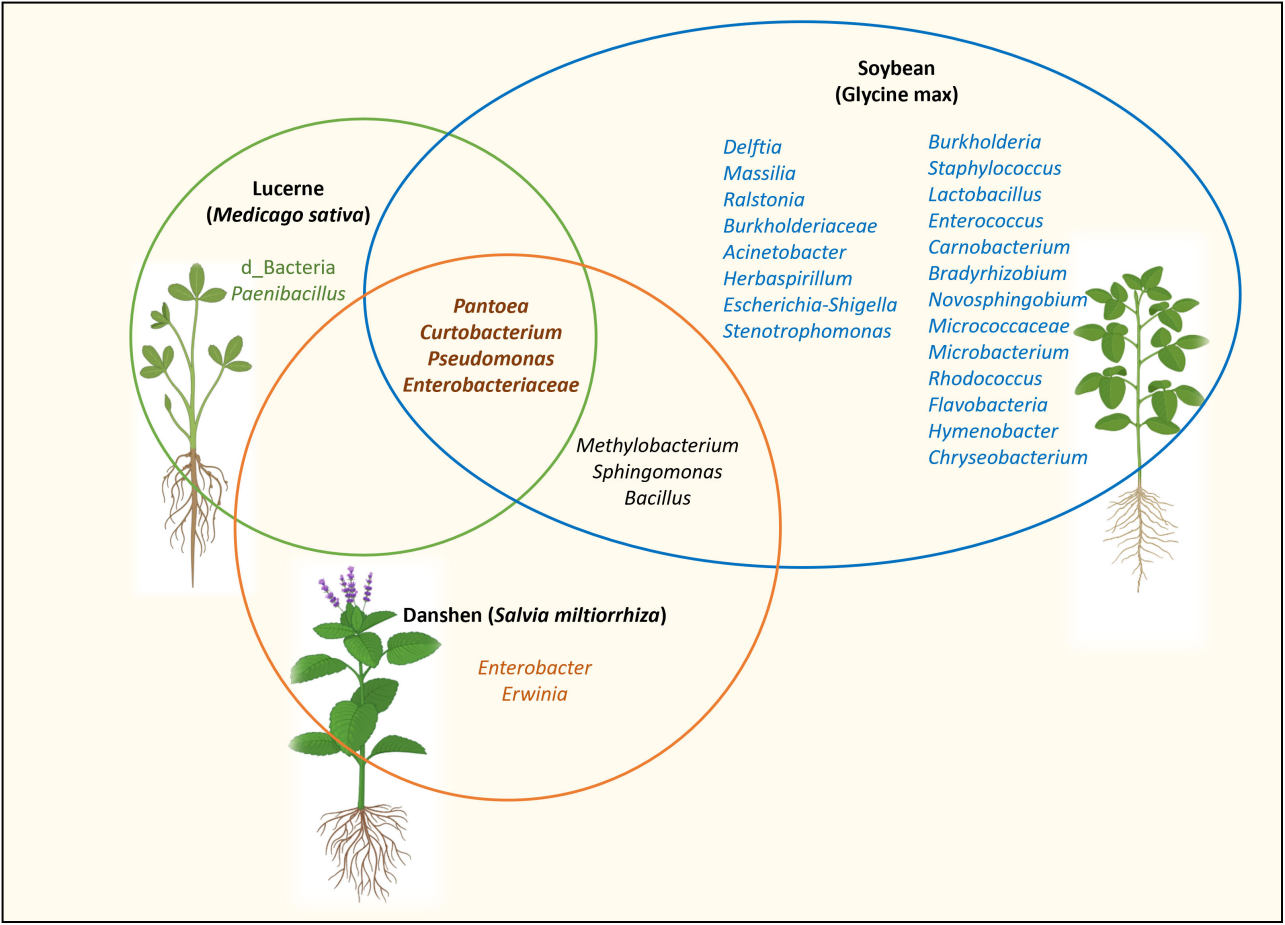
Seed-associated bacterial taxa shared among *Medicago*, soybean and danshen. Soybean bacterial taxa based on Chandel et al. (2022b). Danshen bacterial taxa based on Chen et al. (2018). *Methylobacterium*, *Sphingomonas* and *Bacillus* were identified as core taxa within certain *Medicago* genotypes. However, since they were absent in all *Medicago* genotypes, they were not represented in the *Medicago* core seed microbiome.

In *Medicago*, core taxa included *Pantoea*, *Pseudomonas*, *Paenibacillus*, *Curtobacterium* and members of the Enterobacteriaceae. Similar genera have been reported as core members in the seed microbiomes of soybean (Chandel et al., 2022b) and danshen (*Salvia miltiorrhiza*) (Chen et al., 2018), suggesting a broad ecological relevance (Figure 8). For example, *P. alfalfa*e sp. nov. CQ10 causes bacterial leaf blight in lucerne (Yao et al., 2023), whereas other *Pantoea* strains promote plant growth through phosphate solubilisation, indole-3-acetic acid (IAA) production (Díaz Herrera et al., 2016; Verma et al., 2017), fungal pathogen antagonism (Cottyn et al., 2001; Ruiza et al., 2011), and heavy metal tolerance (Lekired et al., 2023). This functional diversity underscores the importance of validating the ecological role of core taxa at strain level.

*Pseudomonas* spp. includes both pathogenic and beneficial strains. While *P. syringae* causes diseases in crops such as barley, wheat, sugar beet, snap beans and tomato (*Solanum lycopersicum*) (Morris et al., 2007), other strains possess PGP traits including hormone modulation, N fixation, (Goswami et al., 2013; See-Too et al., 2016), and abiotic stress tolerance to drought (Naseem and Bano, 2014), temperature fluctuations (Mishra et al., 2008) and salinity (Saravanakumar and Samiyappan, 2007). In lucerne, co-inoculation with *P. fluorescence* and *R. meliloti* improved growth under salinity stress by enhancing nutrient uptake and reducing sodium accumulation (Younesi et al., 2013).

*Paenibacillus*, a consistently recovered core taxon, is known for key PGP traits including N fixation, phytohormone production, and nutrient solubilisation (Das et al., 2010; Turan et al., 2012; Pandya et al., 2015; Lal et al., 2016). Field trials have shown that strains such as *P. polymyxa* RC05 can enhance wheat yield and soil nutrient availability (Turan et al., 2012), while others offer biocontrol potential against fungal pathogens (DasGupta et al., 2006).

Although some *Curtobacterium* species, such as *C. flaccumfaciens*, are known pathogens, increasing evidence highlights the PGP potential of non-pathogenic strains (Young et al., 1996). Isolates from soil and plant-associated environments have demonstrated traits including systemic resistance induction, enhanced photosynthesis, and improved stress tolerance (Chase et al., 2016). For example, strain ME1 triggered defence responses in cucumber (*Cucumis sativus)* (Raupach and Kloepper, 1998), while *C. albidum* enhanced salt stress resilience in rice (Vimal et al., 2019), underscoring the genus’s biotechnological value for sustainable agriculture.

Several members of Enterobacteriaceae, including *Citrobacter*, *Enterobacter*, *Erwinia*, *Pantoea*, and *Serratia* are well-documented for their PGP properties (Rodríguez-Díaz et al., 2008). *Enterobacter* spp., in particular, contribute to N fixation (James, 2000), siderophore-mediated iron acquisition, hormone production (Maheshwari, 2011), and suppression of seed-borne pathogens such as *Pythium ultimum* (Ryu et al., 2003; Windstam and Nelson Eric, 2008). Some strains also enhance abiotic stress resilience, with *E. asburiae* PS13 conferring cadmium tolerance to mung beans (*Vigna radiata*) (Inouhe et al., 1994), and *E. asburiae* PS2 promoting phosphate solubilisation (Ahemad and Khan, 2010).

Some core taxa were specific and detected at low-abundance, suggesting a genotype-dependent component to the *Medicago* core microbiome. For example, *Bradyrhizobium*, a core member unique to *M. littoralis var. littoralis*, has been reported in other systems to provide N fixation, phosphate solubilisation and phytohormone production (*B. japonicum)* (Cattelan et al., 1999; Boiero et al., 2007; Padukkage et al., 2021).

These findings highlight that even within a conserved core, subtle genotype-specific microbial signatures persist. Profiling the seed microbiomes of *Medicago* CWRs may uncover rare yet functionally important taxa that could contribute to stress resilience, nutrient efficiency, or disease suppression in domesticated lucerne. Further functional validation will be required to determine whether such taxa hold promise for seed-applied microbial consortia or breeding strategies aimed at enhancing crop performance.

### 4.6 Intra-species genomic variation and its implications for strain-level resolution

A diverse assemblage of bacterial species was recovered from *Medicago* seeds, with *Pantoea* emerging as the most abundant and taxonomically diverse genus among the 315 cultured isolates (Supplementary Table 14). While initial species-level identification used 16S rRNA gene sequencing, this marker lacks sufficient resolution to discriminate closely related taxa, even with 98.65–99% similarity thresholds (Kim et al., 2014). To improve resolution, we performed ANI analysis on 34 genome-sequenced isolates against NCBI reference genomes. Using 95–96% ANI threshold (Konstantinidis and Tiedje, 2005; Jain et al., 2018), 12 isolates were resolved to species level and 22 to genus level (Wang et al., 2016).

Pairwise ANI comparisons revealed notable intra-species genomic variation, with ANI scores ranging from 97.85 to 99.99% (Supplementary Table 13), likely reflecting ecological differentiation rather than artefacts (Rodriguez-R et al., 2021). While these findings are based solely on ANI similarity metrics, complementary studies from our group have shown that isolates sharing high genomic similarity can nonetheless exhibit divergent functional outcomes in bioprotection assays (Herath Dissanayakalage et al., 2025a) and SynCom experiments (Herath Dissanayakalage et al., 2025b). This aligns with previous findings of up to 20% gene content divergence between isolates sharing 96.0–99.8% ANI. Some near identical isolates (> 99.99% ANI) still varied by up to 10% in gene content, underscoring the need for more stringent criteria in defining bacterial strains (Rodriguez-R et al., 2024). The term genomovar—describing isolates with >99.5% ANI but distinct profiles (Tomeu et al., 2024)—was adopted for clarity and applied to several groups, including *P. fluorescence* R124 and *P. alli* (Supplementary Table 13). This strain-level resolution has important implications, as isolates belonging to the same species or genomovar may differ in traits relevant to colonisation, persistence, and bioprotection. These insights emphasise the importance of integrating genomic, phenotypic, and ecological validation when evaluating strain-level diversity, particularly in the context of SynCom design, where strain-specific traits rather than species identity alone influence inoculant performance.

### 4.7 Bridging the genotype–phenotype gap: functional unknowns and future directions

While genome-based classification offers powerful resolution, this study highlights a persistent gap between genetic similarity and functional behaviour. Several isolates sharing >99.99% ANI exhibited divergent phenotypic outcomes in bioprotection assays (Herath Dissanayakalage et al., 2025a), reinforcing observations from this and other studies that high genomic similarity does not always translate into equivalent ecological function. This mismatch was especially evident among isolates from CWRs, which often harbour novel genetic elements absent from reference databases. Although annotation tools such as AntiSMASH, dbCAN, and KEGG assist in identifying candidate traits, they provide only partial insights into microbe–host interactions and functional performance in complex environments. To address this genotype-phenotype gap, future studies would benefit from integrating omics approaches with targeted assays. Time-resolved transcriptomics, metabolomics, co-cultivation assays, and SynCom dropout designs offer promising avenues to identify casual relationships between microbial traits and host outcomes.

In parallel, field validation will be essential to evaluate strain persistence, ecological stability, and scalability across environments and host genotypes. Extending this work to other legumes and stress-prone crops may further demonstrate the generalisability of these findings. High-resolution frameworks, such as genomovar-based definitions, are likely to become increasingly important for distinguishing functionally unique strains, facilitating regulatory approval, and guiding microbial inoculant development.

## 5 Conclusion

This study characterised the seed-associated microbiome of lucerne and its CWRs through a dual pipeline that integrated 16S rRNA gene profiling with culture-dependent isolation and genome-level comparative analysis. This approach enabled high-resolution insight into microbial composition, culturability, and intra-species genomic diversity—providing a framework for linking ecological characterisation with functional application. To our knowledge, this represents the first study to characterise seed microbiomes of Australian lucerne and one of the first to compare seed microbiomes of lucerne and its wild relatives using both sequence- and culture-based approaches. The study revealed a conserved core microbiome shared across accessions, alongside species-specific and subdominant taxa shaped by plant genotype and domestication history. Notably, CWRs harboured a broader and more culturable microbial community than domesticated lucerne, reinforcing their value as reservoirs of untapped microbial diversity. Strain-level comparative genomics uncovered extensive intra-species divergence, with ANI-based analyses revealing functional differences even among closely related isolates. These findings emphasise the limitations of taxonomic resolution alone and underscoring the need for strain-resolved resources in microbiome research.

The culture collection developed here has demonstrated translational utility in complementary studies, with selected isolates exhibiting PGP and biocontrol traits, as demonstrated in prior functional studies (Herath Dissanayakalage et al., 2025a; Hone et al., 2025). SynCom experiments using isolates from this library further revealed divergent functional outcomes and confirmed microbial persistence and ecological integration *in planta*, as shown in a companion study—emphasising the complexity of microbiome-host interactions and the importance of strain-level design in microbial applications (Herath Dissanayakalage et al., 2025b). Additional evidence of this utility is provided by a recent study on phosphate solubilising microbes (PSMs), where isolates from this library significantly enhanced P uptake and early lucerne growth (Hone et al., 2025). These cross-study applications reinforce the broader value of curated, genome-characterised collections—not only as taxonomic resources, but as living libraries for trait-guided screening and microbial input development. Collectively, these outcomes highlight the importance of precision in microbiome-informed applications and establish this isolate library as a versatile resource for functional exploration. By integrating culture-based recovery with sequence-based approach, this dual pipeline offers a robust and transferable framework for advancing seed microbiome research across crop systems. Sequence data will be made publicly available upon publication, enabling comparative analyses and reusability. Collectively, this work advances microbiome science from descriptive characterisation to mechanistic insight and application. By establishing a functionally relevant microbial resource and outlining a scalable framework for seed microbiome research, this study lays the foundation for next-generation, precision-designed microbial solutions to support crop resilience, nutrient use efficiency, and sustainable agriculture.

## Data Availability

The full-length 16S sequences of the cultured bacterial strains were deposited in the NCBI GenBank under the BioProject PRJNA1180717.
