# Supplementary material for: Host genotype-driven shifts in the Medicago seed microbiome reveal domestication-linked diversity loss in lucerne (Medicago sativa)
Source: Front Microbiol. 2025 Nov 4;16:1660250. doi: 10.3389/fmicb.2025.1660250 (PMC12623331; doi:10.3389/fmicb.2025.1660250)
Supplement: Supplementary file 1 [file Data_Sheet_1.pdf]

frontiers

# *Supplementary Material*

**Table S2**. Alpha diversity analysis based on Shannon diversity indices for *Medicago* seed samples categorised by host plant species and seed genotypes/accessions

| Category | | | Shannon diversity index |
|---|---|---|---|
| ***Medicago* plant species** | | *M. sativa* L | 1.55 |
| | | *Medicago* crop wild relatives (CWRs) | 2.15 |
| ***Medicago* seed accessions** | *M. sativa* L | Hunter River | 1.72 |
| | | Sequel | 0.97 |
| | | Aurora | 2.37 |
| | | Ryno 6 | 1.74 |
| | | SF Force 5 | 1.93 |
| | | Magna-959 (young seeds) | 2.10 |
| | | Magna-959 (mature seeds) | 1.99 |
| | | Siriver | 2.07 |
| | | Mr Fothergills | 1.56 |
| | | Trifecta | 1.10 |
| | | SARDI 7 Series 2 | 1.52 |
| | | SARDI 10 Series 2 | 1.42 |
| | | Genesis | 1.66 |
| | | SARDI Grazer | 1.45 |
| | | SF 714 | 1.34 |
| | | SF 730 | 1.25 |
| | | SF 914 | 1.32 |
| | | Silverado | 1.40 |
| | *Medicago* CWRs | *Medicago sativa* subsp. *falcata_APG* 6032 | 2.06 |
| | | *Medicago sativa* subsp. *falcata_ APG* 6039 | 1.93 |
| | | *Medicago sativa* subsp. *falcata_ APG* 6925 | 2.03 |
| | | *Medicago littoralis* var. *littoralis_ APG* 20535 | 2.11 |
| | | *Medicago littoralis* var. *littoralis_ APG* 21384 | 1.96 |
| | | *Medicago littoralis* var. *littoralis_ APG* 21559 | 1.95 |
| | | *Medicago littoralis* var. *littoralis_ APG* 32892 | 1.87 |
| | | *Medicago laciniata_ APG* 21164 | 2.57 |
| | | *Medicago laciniata_ APG* 21177 | 2.43 |
| | | *Medicago laciniata_ APG* 20841 | 2.47 |
| | | *Medicago laciniata_ APG* 21700 | 2.41 |
| | | *Medicago truncatula_ APG* 20935 | 2.41 |
| | | *Medicago truncatula_ APG* 21758 | 2.45 |
| | | *Medicago truncatula_ APG* 21771 | 2.44 |
| | | *Medicago littoralis_ APG* 21177 | 2.09 |
| | | *Medicago littoralis_ APG* 21198 | 1.82 |
| | | *Medicago littoralis_ APG* 21232 | 1.96 |
| | | *Medicago littoralis_ APG* 21235 | 1.73 |

frontiers

**Table S3**. The Kruskal-Wallis pairwise statistical test demonstrating the significant differences in bacterial species richness when grouped based on *Medicago* plant species. *p* values < 0.05 are highlighted in bold.

| ***Medicago* species** | | ***p*-value** |
|---|---|---|
| **Group 1** | **Group 2** | |
| *M. sativa* L | *Medicago* CWRs | **1.16E-29** |
| *M. laciniata* | *M. littoralis* | **1.74E-10** |
| *M. laciniata* | *M. littoralis* var. *littoralis* | **1.53E-10** |
| *M. laciniata* | *M. sativa* L | **2.86E-29** |
| *M. laciniata* | *M. sativa* subsp. *falcata* | **0.007419** |
| *M. laciniata* | *M. truncatula* | 0.993698 |
| *M. littoralis* | *M. littoralis* var. *littoralis* | 0.575331 |
| *M. littoralis* | *M. sativa* L | **1.05E-05** |
| *M. littoralis* | *M. sativa* subsp. *falcata* | 0.130706 |
| *M. littoralis* | *M. truncatula* | **9.49E-07** |
| *M. littoralis* var. *littoralis* | *M. sativa* L | **4.82E-08** |
| *M. littoralis* var. *littoralis* | *M. sativa* subsp. *falcata* | 0.297164 |
| *M. littoralis* var. *littoralis* | *M. truncatula* | **3.48E-06** |
| *M. sativa* L | *M. sativa* subsp. *falcata* | **1.21E-05** |
| *M. sativa* L | *M. truncatula* | **1.82E-16** |
| *M. sativa* subsp. *falcata* | *M. truncatula* | **0.022632** |

**Table S4**. Differences in bacterial microbiome composition calculated based on *Medicago* species using PERMANOVA and PERMDISP values obtained from unweighted Unifrac distance matrix PCoA data. *p* values < 0.5 are highlighted in bold.

| ***Medicago* species** | | **PERMANOVA** | **PERMDISP** |
|---|---|---|---|
| | | **Unweighted** | **Unweighted** |
| **Group 1** | **Group 2** | ***p* value** | ***p* value** |
| *M. laciniata* | *M. littoralis* | **0.01** | 0.055 |
| *M. laciniata* | *M. littoralis* var. *littoralis* | **0.01** | 0.189 |
| *M. laciniata* | *M. sativa* L | **0.01** | **0.001** |
| *M. laciniata* | *M. sativa* subsp. *falcata* | **0.01** | 0.978 |
| *M. laciniata* | *M. truncatula* | **0.01** | **0.003** |
| *M. littoralis* | *M. littoralis* var. *littoralis* | **0.01** | **0.004** |
| *M. littoralis* | *M. sativa* L | **0.01** | **0.007** |
| *M. littoralis* | *M. sativa* subsp. *falcata* | **0.01** | 0.148 |
| *M. littoralis* | *M. truncatula* | **0.01** | **0.001** |
| *M. littoralis* var. *littoralis* | *M. sativa* L | **0.01** | **0.001** |
| *M. littoralis* var. *littoralis* | *M. sativa* subsp. *falcata* | **0.01** | 0.219 |
| *M. littoralis* var. *littoralis* | *M. truncatula* | **0.01** | 0.121 |
| *M. sativa* L | *M. sativa* subsp. *falcata* | **0.01** | **0.001** |
| *M. sativa* L | *M. truncatula* | **0.01** | **0.001** |
| *M. sativa* subsp. *falcata* | *M. truncatula* | **0.01** | **0.001** |

frontiers

**Table S5**. Relative ASV abundance of bacterial communities associated with *Medicago* seed microbiome at phylum level classified based on host plant species

| Phylum | Relative ASV abundance of bacterial phyla (%) | |
|---|---|---|
| | Domesticated lucerne seed accessions | *Medicago* CWR seed accessions |
| Proteobacteria | 77.635% | 77.999% |
| Firmicutes | 21.568% | 20.517% |
| Actinobacteriota | 0.757% | 1.072% |
| Bacteroidota | 0.002% | 0.397% |
| Deinococcota | 0.000% | 0.000% |
| Patescibacteria | 0.000% | 0.000% |
| Planctomycetota | 0.000% | 0.000% |
| Unassigned | 0.034% | 0.015% |
| Verrucomicrobiota | 0.004% | 0.000% |

frontiers

**Table S6**. Relative ASV abundance of bacterial communities associated with *Medicago* seed microbiome at phylum level classified based on host plant.

| *Medicago* seed accession | Relative abundance of bacterial phyla | | | | | | | | |
|---|---|---|---|---|---|---|---|---|---|
| | Proteobacteria | Firmicutes | Actinobacteriota | Bacteroidota | Verrucomicrobiota | Patescibacteria | Deinococcota | Planctomycetota | Unassigned |
| Hunter river | 97.603% | 1.278% | 0.520% | 0.040% | 0.181% | 0.000% | 0.000% | 0.000% | 0.379% |
| Sequel | 99.945% | 0.005% | 0.049% | 0.000% | 0.000% | 0.000% | 0.000% | 0.000% | 0.001% |
| Trifecta | 99.585% | 0.237% | 0.158% | 0.005% | 0.000% | 0.000% | 0.000% | 0.000% | 0.015% |
| Mr Fothergills | 97.301% | 1.505% | 0.708% | 0.023% | 0.011% | 0.000% | 0.000% | 0.000% | 0.452% |
| Aurora | 53.850% | 45.936% | 0.186% | 0.000% | 0.000% | 0.000% | 0.000% | 0.000% | 0.028% |
| SF Force 5 | 63.289% | 36.596% | 0.109% | 0.000% | 0.000% | 0.000% | 0.000% | 0.000% | 0.007% |
| Ryno 6 | 63.163% | 36.645% | 0.186% | 0.001% | 0.001% | 0.000% | 0.000% | 0.001% | 0.003% |
| Siriver | 49.674% | 50.026% | 0.271% | 0.000% | 0.000% | 0.000% | 0.000% | 0.002% | 0.028% |
| Magna-959 (young seeds) | 62.598% | 37.083% | 0.306% | 0.001% | 0.003% | 0.000% | 0.000% | 0.000% | 0.009% |
| Magna-959 (mature seeds) | 58.893% | 40.946% | 0.144% | 0.002% | 0.000% | 0.000% | 0.000% | 0.000% | 0.014% |
| SARDI 7 Series 2 | 67.817% | 31.835% | 0.319% | 0.000% | 0.000% | 0.000% | 0.000% | 0.000% | 0.029% |
| Genesis | 71.452% | 28.003% | 0.515% | 0.000% | 0.002% | 0.000% | 0.000% | 0.000% | 0.029% |
| SARDI Grazer | 93.881% | 5.020% | 1.084% | 0.000% | 0.001% | 0.000% | 0.000% | 0.000% | 0.014% |
| SARDI 10 Series 2 | 67.856% | 31.205% | 0.887% | 0.003% | 0.002% | 0.000% | 0.000% | 0.000% | 0.046% |
| Silverado | 72.279% | 27.224% | 0.460% | 0.003% | 0.001% | 0.000% | 0.000% | 0.000% | 0.034% |
| SF 714 | 95.199% | 3.189% | 1.594% | 0.000% | 0.000% | 0.000% | 0.001% | 0.000% | 0.018% |
| SF 730 | 93.638% | 4.427% | 1.929% | 0.000% | 0.000% | 0.000% | 0.000% | 0.000% | 0.007% |
| SF 914 | 92.373% | 5.276% | 2.341% | 0.000% | 0.001% | 0.000% | 0.000% | 0.000% | 0.009% |
| *M.sativa* subsp. *falcata*-APG 6032 | 54.335% | 45.235% | 0.424% | 0.004% | 0.000% | 0.000% | 0.000% | 0.000% | 0.002% |
| *M. sativa* subsp. *falcata*-APG 6925 | 46.311% | 53.217% | 0.469% | 0.000% | 0.000% | 0.000% | 0.000% | 0.000% | 0.003% |
| *M.sativa* subsp. *falcata*-APG 6039 | 49.520% | 49.993% | 0.484% | 0.000% | 0.001% | 0.000% | 0.000% | 0.000% | 0.001% |
| *M. littoralis*- APG 21177 | 65.214% | 34.433% | 0.030% | 0.314% | 0.002% | 0.000% | 0.000% | 0.000% | 0.007% |

| | | | | | | | | | |
|---|---|---|---|---|---|---|---|---|---|
| *M. littoralis*- APG 21235 | 80.106% | 19.824% | 0.062% | 0.001% | 0.000% | 0.000% | 0.000% | 0.000% | 0.007% |
| *M. littoralis*- APG 21198 | 71.844% | 27.767% | 0.375% | 0.003% | 0.000% | 0.000% | 0.000% | 0.000% | 0.011% |
| *M. littoralis*- APG 21232 | 75.865% | 23.707% | 0.410% | 0.009% | 0.001% | 0.000% | 0.000% | 0.000% | 0.008% |
| *M. littoralis* var. *littoralis*- APG 20535 | 85.331% | 14.127% | 0.487% | 0.049% | 0.001% | 0.000% | 0.000% | 0.000% | 0.007% |
| *M. littoralis* var. *littoralis*- APG 32892 | 87.372% | 12.008% | 0.613% | 0.002% | 0.000% | 0.001% | 0.000% | 0.000% | 0.003% |
| *M. littoralis* var. *littoralis*- APG 21384 | 84.568% | 14.952% | 0.473% | 0.004% | 0.000% | 0.001% | 0.000% | 0.000% | 0.002% |
| *M. littoralis* var. *littoralis*- APG 21559 | 85.966% | 13.144% | 0.874% | 0.007% | 0.000% | 0.000% | 0.000% | 0.000% | 0.009% |
| *M. laciniata*- APG 20841 | 85.587% | 11.165% | 2.397% | 0.797% | 0.000% | 0.000% | 0.000% | 0.000% | 0.053% |
| *M. laciniata*- APG 21177 | 83.864% | 11.035% | 3.352% | 1.705% | 0.000% | 0.000% | 0.000% | 0.000% | 0.044% |
| *M. laciniata*- APG 21700 | 86.674% | 9.952% | 2.372% | 0.978% | 0.000% | 0.000% | 0.000% | 0.000% | 0.024% |
| *M. laciniata*- APG 21164 | 83.061% | 12.665% | 2.159% | 2.057% | 0.000% | 0.000% | 0.000% | 0.000% | 0.059% |
| *M. trancatula*- APG 20935 | 90.314% | 8.389% | 1.283% | 0.013% | 0.000% | 0.000% | 0.000% | 0.000% | 0.001% |
| *M. trancatula*- APG 21771 | 88.445% | 10.004% | 1.243% | 0.306% | 0.000% | 0.000% | 0.000% | 0.000% | 0.002% |
| *M. trancatula*- APG 21758 | 87.457% | 10.438% | 1.591% | 0.513% | 0.000% | 0.000% | 0.000% | 0.000% | 0.001% |

**Table S7**. Relative ASV abundance of bacterial communities associated with *Medicago* seed microbiome at class level classified based on host plant species

| Class | Relative abundance of bacterial classes (%) | |
|---|---|---|
| | Domesticated lucerne seed accessions | *Medicago* CWR seed accessions |
| Gammaproteobacteria | 77.193% | 75.036% |
| Bacilli | 21.567% | 20.517% |
| Actinobacteria | 0.757% | 1.072% |
| Alphaproteobacteria | 0.442% | 2.963% |
| Unassigned | 0.034% | 0.015% |
| Verrucomicrobiae | 0.004% | 0.000% |
| Bacteroidia | 0.002% | 0.397% |
| Clostridia | 0.001% | 0.000% |
| Planctomycetes | 0.000% | 0.000% |
| Deinococci | 0.000% | 0.000% |
| Saccharimonadia | 0.000% | 0.000% |
| Thermoleophilia | 0.000% | 0.000% |

frontiers

**Table S8**. Relative ASV abundance of bacterial communities associated with *Medicago* seed microbiome at class level classified based on seed accessions.

| Class | *Gammaproteobacteria* | *Actinobacteria* | *Bacteroidia* | *Bacilli* | *Saccharimonadia* | *Alphaproteobacteria* | *Verrucomicrobiae* | *Clostridia* | *Thermoleophilia* | *Deinococci* | Unassigned | *Planctomycetes* |
|---|---|---|---|---|---|---|---|---|---|---|---|---|
| Hunter River | 96.822% | 0.520% | 0.040% | 1.235% | 0.000% | 0.780% | 0.181% | 0.043% | 0.000% | 0.000% | 0.379% | 0.000% |
| Sequel | 99.942% | 0.049% | 0.000% | 0.005% | 0.000% | 0.003% | 0.000% | 0.000% | 0.000% | 0.000% | 0.001% | 0.000% |
| Aurora | 53.845% | 0.186% | 0.000% | 45.936% | 0.000% | 0.005% | 0.000% | 0.000% | 0.000% | 0.000% | 0.028% | 0.000% |
| Ryno 6 | 63.128% | 0.186% | 0.001% | 36.645% | 0.000% | 0.035% | 0.001% | 0.000% | 0.000% | 0.000% | 0.003% | 0.001% |
| SF Force-5 | 63.256% | 0.109% | 0.000% | 36.596% | 0.000% | 0.033% | 0.000% | 0.000% | 0.000% | 0.000% | 0.007% | 0.000% |
| Magna-959 (young seeds) | 62.538% | 0.306% | 0.001% | 37.083% | 0.000% | 0.060% | 0.003% | 0.000% | 0.000% | 0.000% | 0.009% | 0.000% |
| Magna-959 (mature seeds) | 58.866% | 0.144% | 0.002% | 40.946% | 0.000% | 0.028% | 0.000% | 0.000% | 0.000% | 0.000% | 0.014% | 0.000% |
| Siriver | 49.674% | 0.271% | 0.000% | 50.026% | 0.000% | 0.000% | 0.000% | 0.000% | 0.000% | 0.000% | 0.028% | 0.002% |
| Fothergill | 81.178% | 0.708% | 0.023% | 1.505% | 0.000% | 16.123% | 0.011% | 0.000% | 0.000% | 0.000% | 0.452% | 0.000% |
| Trifecta | 99.481% | 0.158% | 0.005% | 0.237% | 0.000% | 0.104% | 0.000% | 0.000% | 0.000% | 0.000% | 0.015% | 0.000% |
| SARDI 7 Series 2 | 67.636% | 0.319% | 0.000% | 31.835% | 0.000% | 0.181% | 0.000% | 0.000% | 0.000% | 0.000% | 0.029% | 0.000% |
| SARDI 10 Series 2 | 67.833% | 0.887% | 0.003% | 31.205% | 0.000% | 0.023% | 0.002% | 0.000% | 0.000% | 0.000% | 0.046% | 0.000% |
| Genesis | 71.406% | 0.515% | 0.000% | 28.003% | 0.000% | 0.047% | 0.002% | 0.000% | 0.000% | 0.000% | 0.029% | 0.000% |
| SARDI Grazer | 93.649% | 1.084% | 0.000% | 5.020% | 0.000% | 0.232% | 0.001% | 0.000% | 0.000% | 0.000% | 0.014% | 0.000% |
| SF 714 | 95.093% | 1.594% | 0.000% | 3.189% | 0.000% | 0.106% | 0.000% | 0.000% | 0.000% | 0.001% | 0.018% | 0.000% |
| SF 730 | 93.581% | 1.929% | 0.000% | 4.427% | 0.000% | 0.057% | 0.000% | 0.000% | 0.000% | 0.000% | 0.007% | 0.000% |
| SF 914 | 92.158% | 2.341% | 0.000% | 5.276% | 0.000% | 0.215% | 0.001% | 0.000% | 0.000% | 0.000% | 0.009% | 0.000% |
| Silverado | 72.222% | 0.460% | 0.003% | 27.224% | 0.000% | 0.058% | 0.001% | 0.000% | 0.000% | 0.000% | 0.034% | 0.000% |
| *M. sativa* subsp. *falcata*- APG 6032 | 52.434% | 0.424% | 0.004% | 45.235% | 0.000% | 1.901% | 0.000% | 0.000% | 0.000% | 0.000% | 0.002% | 0.000% |

| | | | | | | | | | | | | |
|---|---|---|---|---|---|---|---|---|---|---|---|---|
| *M. sativa* subsp. *falcata*- APG 6039 | 47.795% | 0.484% | 0.000% | 49.993% | 0.000% | 1.726% | 0.001% | 0.000% | 0.000% | 0.000% | 0.001% | 0.000% |
| *M. sativa* subsp. *falcata*- APG 6925 | 44.689% | 0.465% | 0.000% | 53.217% | 0.000% | 1.621% | 0.000% | 0.000% | 0.003% | 0.000% | 0.003% | 0.000% |
| *M. littoralis*- APG 21177 | 64.677% | 0.030% | 0.314% | 34.432% | 0.000% | 0.538% | 0.002% | 0.001% | 0.000% | 0.000% | 0.007% | 0.000% |
| *M. littoralis* - APG 21235 | 79.994% | 0.062% | 0.001% | 19.823% | 0.000% | 0.112% | 0.000% | 0.001% | 0.000% | 0.000% | 0.007% | 0.000% |
| *M. littoralis* - APG 21198 | 71.472% | 0.375% | 0.003% | 27.767% | 0.000% | 0.372% | 0.000% | 0.000% | 0.000% | 0.000% | 0.011% | 0.000% |
| *M. littoralis* - APG 21232 | 75.386% | 0.410% | 0.009% | 23.707% | 0.000% | 0.479% | 0.001% | 0.000% | 0.000% | 0.000% | 0.008% | 0.000% |
| *M. littoralis* var. *littoralis*- APG 20535 | 78.801% | 0.487% | 0.049% | 14.127% | 0.000% | 6.530% | 0.001% | 0.000% | 0.000% | 0.000% | 0.007% | 0.000% |
| *M. littoralis* var. *littoralis* - APG 32892 | 81.708% | 0.613% | 0.002% | 12.008% | 0.001% | 5.665% | 0.000% | 0.000% | 0.000% | 0.000% | 0.003% | 0.000% |
| *M. littoralis* var. *littoralis* - APG 21384 | 77.006% | 0.473% | 0.004% | 14.952% | 0.001% | 7.562% | 0.000% | 0.000% | 0.000% | 0.000% | 0.002% | 0.000% |
| *M. littoralis* var. *littoralis* APG -21559 | 79.049% | 0.874% | 0.007% | 13.144% | 0.000% | 6.917% | 0.000% | 0.000% | 0.000% | 0.000% | 0.009% | 0.000% |
| *M. laciniata*- APG 20841 | 82.386% | 2.397% | 0.797% | 11.165% | 0.000% | 3.202% | 0.000% | 0.000% | 0.000% | 0.000% | 0.053% | 0.000% |
| *M. laciniata* - APG 21700 | 84.131% | 2.372% | 0.978% | 9.952% | 0.000% | 2.543% | 0.000% | 0.000% | 0.000% | 0.000% | 0.024% | 0.000% |
| *M. laciniata* - APG 21164 | 79.288% | 2.159% | 2.057% | 12.665% | 0.000% | 3.773% | 0.000% | 0.000% | 0.000% | 0.000% | 0.059% | 0.000% |
| *M. laciniata* - APG 21177 | 81.863% | 3.352% | 1.705% | 11.035% | 0.000% | 2.001% | 0.000% | 0.000% | 0.000% | 0.000% | 0.044% | 0.000% |
| *M. trancatula*- APG 21771 | 85.761% | 1.243% | 0.306% | 10.004% | 0.000% | 2.684% | 0.000% | 0.000% | 0.000% | 0.000% | 0.002% | 0.000% |
| *M. trancatula* - APG 20935 | 88.339% | 1.283% | 0.013% | 8.389% | 0.000% | 1.975% | 0.000% | 0.000% | 0.000% | 0.000% | 0.001% | 0.000% |
| *M. trancatula* - APG 21758 | 84.974% | 1.591% | 0.513% | 10.438% | 0.000% | 2.483% | 0.000% | 0.000% | 0.000% | 0.000% | 0.001% | 0.000% |

**Table S9**. Relative ASV abundance of bacterial communities associated with *Medicago* seed microbiome at genus level classified based on host species.

| **Genus** | **Domesticated lucerne seed accessions** | ***Medicago* CWR seed accessions** |
|---|---|---|
| *g__Pantoea* | 0.536 | 0.311 |
| *g__Paenibacillus* | 0.206 | 0.198 |
| *g__Pseudomonas* | 0.166 | 0.273 |
| *f__*Enterobacteriaceae | 0.040 | 0.024 |
| *f__*Erwiniaceae | 0.018 | 0.000 |
| *g__Massilia* | 0.010 | 0.089 |
| *g__Bacillus* | 0.008 | 0.004 |
| *g__Curtobacterium* | 0.007 | 0.010 |
| *g__Methylobacterium-Methylorubrum* | 0.004 | 0.008 |
| *g__Saccharibacillus* | 0.001 | 0.002 |
| *g__Xanthomonas* | 0.001 | 0.000 |
| Unassigned | 0.001 | 0.000 |
| *g__Sphingomonas* | 0.001 | 0.017 |
| *o*__Enterobacterales | 0.001 | 0.000 |
| Others | 0.000 | 0.000 |
| *g__Uliginosibacterium* | 0.000 | 0.000 |
| *g__Anaerobacillus* | 0.000 | 0.000 |
| *f__*Comamonadaceae | 0.000 | 0.003 |
| *g__Burkholderia-Caballeronia-Paraburkholderia* | 0.000 | 0.000 |
| *g__Stenotrophomonas* | 0.000 | 0.002 |
| *g__Allorhizobium-Neorhizobium-Pararhizobium-Rhizobium* | 0.000 | 0.003 |
| *g__Tumebacillus* | 0.000 | 0.001 |
| *f__*Microbacteriaceae | 0.000 | 0.000 |
| *f__*Oxalobacteraceae | 0.000 | 0.001 |
| *g__Kineococcus* | 0.000 | 0.000 |
| *g__Duganella* | 0.000 | 0.046 |
| *g__Hymenobacter* | 0.000 | 0.003 |
| *g__Advenella* | 0.000 | 0.001 |
| *g__Novosphingobium* | 0.000 | 0.000 |
| *g__Pedobacter* | 0.000 | 0.000 |
| *g__Chryseobacterium* | 0.000 | 0.000 |
| *g__Plantibacter* | 0.000 | 0.000 |
| *g__Variovorax* | 0.000 | 0.000 |
| *g__Sanguibacter* | 0.000 | 0.000 |

frontiers

**Table S10.** Relative abundance of core bacterial taxa shared among all *Medicago* species and unique taxa for each plant species, including the percentage of bacterial taxa exclusive to the core microbiomes of each plant species.

| | Class | Genus | *M. sativa* | *M. sativa* subsp. *falcata* | *M. laciniata* | *M. littoralis* | *M. littoralis* var. *littoralis* | *M. truncatula* |
|---|---|---|---|---|---|---|---|---|
| Bacterial core taxa shared by all six *Medicago* species | *d*__Bacteria | *d*__Bacteria | 0.034 | 0.002 | 0.045 | 0.008 | 0.005 | 0.001 |
| | Gammaproteobacteria | *g__Pseudomonas* | 16.586 | 16.02 | 21.687 | 29.982 | 33.201 | 32.958 |
| | Gammaproteobacteria | *f*__Enterobacteriaceae | 3.964 | 1.334 | 2.415 | 3.372 | 2.327 | 1.75 |
| | Gammaproteobacteria | *g__Pantoea* | 53.644 | 21.87 | 39.478 | 26.136 | 30.786 | 35.117 |
| | Actinobacteria | *g__Curtobacterium* | 0.728 | 0.446 | 2.464 | 0.214 | 0.595 | 1.098 |
| | Bacilli | *g__Paenibacillus* | 20.633 | 47.368 | 10.653 | 25.586 | 13.567 | 9.013 |
| Percentage of bacterial taxa exclusive to the plant's core microbiome | | | 0.000 | 1.281 | 0.137 | 0.000 | 0.119 | 0.536 |

**Table S11.** Relative abundance of core bacterial taxa shared between domesticated lucerne and *Medicago* CWR seeds, including the percentage of bacterial taxa exclusive to the core microbiomes of each plant species.

| | Class | Genus | Domesticated lucerne | *Medicago* CWRs |
|---|---|---|---|---|
| Bacterial core taxa shared by *M. sativa* and *Medicago* CWRs | d__*Bacteria* | d__*Bacteria* | 0.02 | 0.01 |
| | *Gammaproteobacteria* | g__*Pseudomonas* | 8.50 | 17.04 |
| | *Gammaproteobacteria* | f__*Enterobacteriaceae* | 2.03 | 1.49 |
| | *Gammaproteobacteria* | g__*Pantoea* | 27.50 | 19.46 |
| | *Actinobacteria* | g__*Curtobacterium* | 0.37 | 0.63 |
| | *Bacilli* | g__*Paenibacillus* | 10.58 | 12.38 |

**Table S12**. Closest taxonomy IDs identified by Kraken database for 34 bacterial isolates. ANI values > 95.00% are highlighted in bold. Genome sequences for bacterial isolates are described under NCBI

| Sample ID | Closest taxonomy ID identified by Kraken database | Host plant species | ANI | |
|---|---|---|---|---|
| | | | ANI% | NCBI reference genome ID |
| Lu_LA164_018 | *Pseudomonas orientalis* | *M. laciniata* | 94.58% | *Pseudomonas orientalis* - *GCF_003852045.1* |
| Lu_MgY_007 | *Paenibacillus* sp. IHB B 3084 | *M. sativa* | 94.74% | *Paenibacillus* sp. IHB B 3084 - GCF_001447315.1 |
| Lu_LT198_010 | *Pseudomonas* sp. Leaf127 | *M. littoralis* | 91.76% | *Pseudomonas* sp. Leaf127 - GCF_001423155.1 |
| Lu_LA164_012 | *Pseudomonas graminis* | *M. laciniata* | 85.93% | *Pseudomonas graminis* DSM 11363 - GCF_900111735.1 |
| Lu_LA164_009 | *Curtobacterium* sp. VKM Ac-2852 | *M. laciniata* | 97.55% | *Curtobacterium* sp. VKM Ac-2852 - GCF_013205045.1 |
| Lu_TR758_W005 | *Pantoea varia* | *M. trancatula* | 97.26% | *Pantoea varia* -GCF_900115075.1 |
| Lu_Au_053 | *Enterobacter* sp. | *M. sativa* | NA | |
| Lu_TR758_015 | *Pantoea allii* | *M. trancatula* | 80.47% | *Pantoea allii* - GCF_003148935.1 |
| Lu_F5_006 | *Pseudomonas fluorescens* R124 | *M. sativa* | 81.80% | *Pseudomonas fluorescens* IMG-taxon 2617270901 annotated assembly - GCF_900215245.1 |
| Lu_LA841_009 | *Pantoea allii* | *M. laciniata* | 99.80% | *Pantoea allii* - GCF_003148935.1 |
| Lu_TR758_007 | *Pantoea allii* | *M. trancatula* | 80.48% | *Pantoea allii* - GCF_003148935.1 |
| Lu_LT198_003 | *Pantoea agglomerans* pv. *betae* | *M. littoralis* | 97.48% | *Pantoea* agglomerans - GCF_019048385.1) |
| Lu_LT198_W003 | *Pseudomonas graminis* | *M. littoralis* | 99.85% | *Pseudomonas graminis DSM 11363* - GCF_900111735.1 |
| Lu_LT198_018 | *Pantoea allii* | *M. littoralis* | 80.43% | *Pantoea allii* - GCF_003148935.1 |
| Lu_LA841_007 | *Pantoea allii* | *M. laciniata* | 80.43% | *Pantoea allii* - GCF_003148935.1 |
| Lu_F5_029 | *Pseudomonas fluorescens* R124 | *M. sativa* | 81.81% | *Pseudomonas fluorescens* IMG-taxon 2617270901 annotated assembly - GCF_900215245.1 |
| Lu_LT235_004 | *Pantoea sp.* | *M. littoralis* | NA | |
| Lu_R6_023 | *Duffyella gerundensis* | *M. sativa* | 98.82% | *Duffyella gerundensis* - GCF_020342335.1 |
| Lu_LT198_002 | *Pantoea allii* | *M. littoralis* | 80.43% | *Pantoea allii* - GCF_003148935.1 |
| Lu_Au_058 | *Kosakonia cowanii* JCM 10956 = DSM 18146 | *M. sativa* | 98.28% | *Kosakonia cowanii* - GCF_001975225.1 |
| Lu_TR771_006 | *Pantoea agglomerans* Eh318 | *M. trancatula* | 97.40% | *Pantoea agglomerans* - GCF_000687245.1 |
| Lu_LT177_010 | *Paenibacillus* sp. JNUCC-31 | *M. trancatula* | 86.87% | *Paenibacillus* sp. JNUCC-31 - GCF_014844075.1 |
| Lu_LA700_W009 | *Pantoea* sp. | *M. laciniata* | NA | |

| | | | | |
|---|---|---|---|---|
| Lu_LA164_003 | *Pantoea agglomerans* pv. *betae* | *M. laciniata* | 98.53% | *Pantoea agglomerans* - GCF_019048385.1 |
| Lu_Lu_F5_028 | *Duffyella gerundensis* | *M. sativa* | 98.97% | *Duffyella gerundensis* - GCF_020342335.1 |
| Lu_LA841_015 | *Pseudomonas* sp. KU26590 | *M. laciniata* | 85.94% | *Pseudomonas* sp. KU26590 - GCF_026153515.1 |
| Lu_TR935_010 | *Duganella zoogloeoides* | *M. littoralis* | 89.99% | *Duganella zoogloeoides* - GCF_034479515.1 |
| Lu_Sv_042 | *Paenibacillus amylolyticus* | *M. sativa* | 96.46% | *Paenibacillus amylolyticus* - GCF_029542105.1 |
| Lu_F5_008 | *Massilia* sp. | *M. sativa* | NA | |
| Lu_TR758_011 | *Pseudomonas* sp. KU26590 | *M. trancatula* | 86.02% | *Pseudomonas* sp. KU26590 - GCF_026153515.1 |
| Lu_LT198_042 | *Paenibacillus nuruki* | *M. littoralis* | 98.42% | *Paenibacillus nuruki* - GCF_963311185.1 |
| Lu_TR771_007 | *Paenibacillus nuruki* | *M. trancatula* | 98.54% | *Paenibacillus nuruki* - GCF_963311185.1 |
| Lu_TR758_008 | *Paenibacillus* sp. KACC 21273 | *M. trancatula* | 86.75% | *Paenibacillus* sp. KACC 21273 - GCF_028736095.1 |
| Lu_TR935_014 | *Pseudomonas viridiflava* CC1582 | M. trancatula | 96.60% | *Pseudomonas viridiflava* CC1582 - GCF_000452505.1 |

**S13**. Pairwise ANI comparisons between the closely related isolates of the same species.

| **Reference genome** | **Query Genome** | **ANI%** |
|---|---|---|
| *Pseudomonas fluorescence* R124 | | |
| Lu_F5_029 | Lu_F5_006 | 99.99% |
| *Duffyella gerundiensis* | | |
| Lu_F5_028 | Lu_R6_023 | 98.85% |
| *Pseudomonas* sp. KU26590 | | |
| Lu_TR758_011 | Lu_LA841_015 | 98.44% |
| *Pantoea alli* | | |
| Lu_TR758_007 | Lu_TR758_015 | 99.99% |
| Lu_LT198_018 | Lu_TR758_015 | 99.54% |
| Lu_LT198_018 | Lu_TR758_007 | 99.54% |
| LA841_007 | Lu_TR758_015 | 99.55% |
| LA841_007 | Lu_TR758_007 | 99.54% |
| LA841_007 | Lu_LT198_018 | 99.81% |
| Lu_LT198_002 | Lu_TR758_015 | 99.54% |
| Lu_LT198_002 | Lu_TR758_007 | 99.53% |
| Lu_LT198_002 | Lu_LT198_018 | 99.99% |
| Lu_LT198_002 | Lu_LA841_007 | 99.80% |
| Lu_LA841_009 | Lu_TR758_015 | 99.53% |
| Lu_LA841_009 | Lu_TR758_007 | 99.55% |
| Lu_LA841_009 | Lu_LT198_018 | 99.75% |
| Lu_LA841_009 | Lu_LA841_007 | 99.92% |
| Lu_LA841_009 | Lu_LT198_002 | 99.75% |
| *Paenibacillus nuruki* | | |
| Lu_TR771_007 | Lu_LT198_042 | 98.50% |

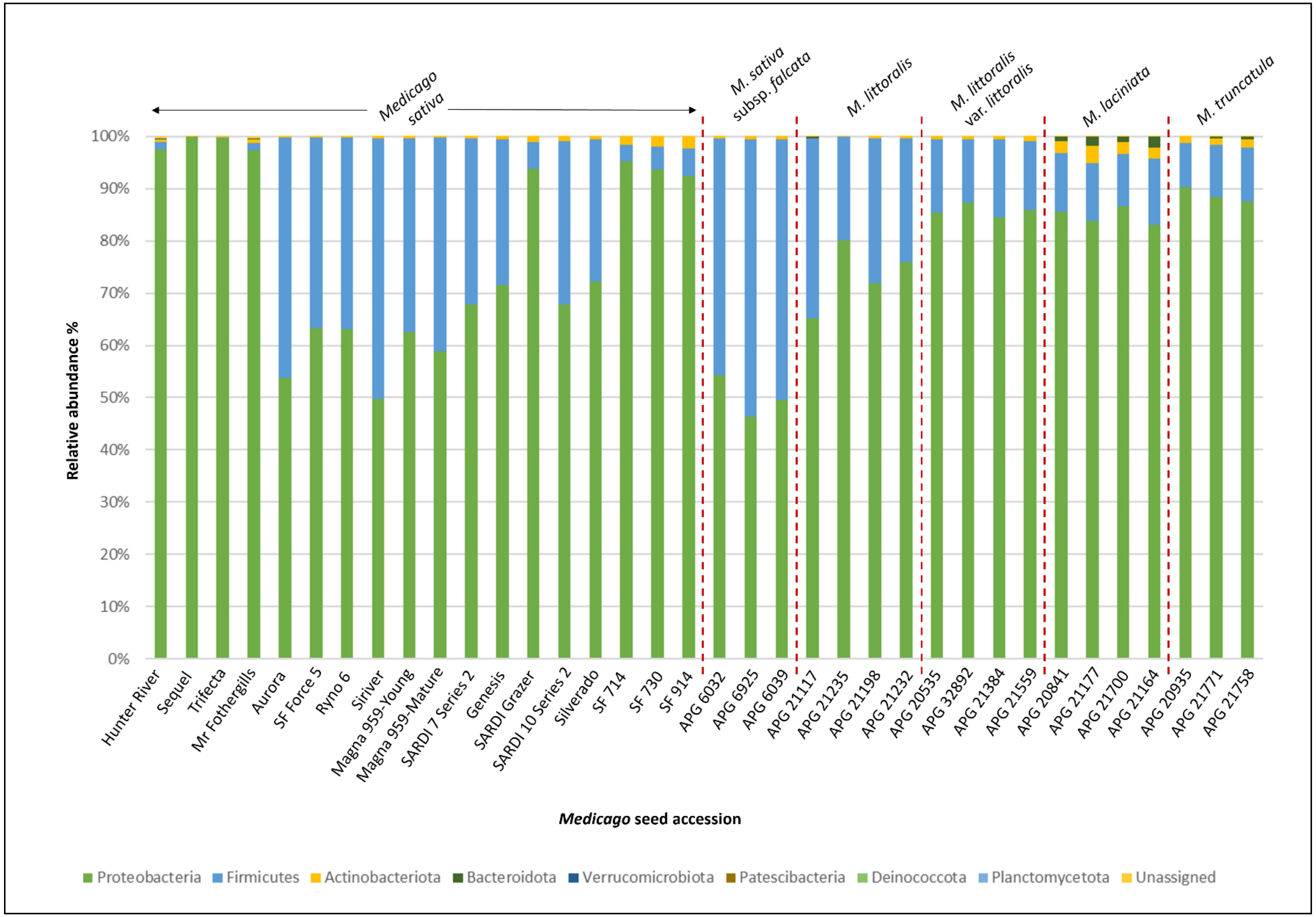


**Figure S1**. Relative ASV abundance of bacterial communities associated with *Medicago* seed microbiome at phylum level classified based on *Medicago* host species

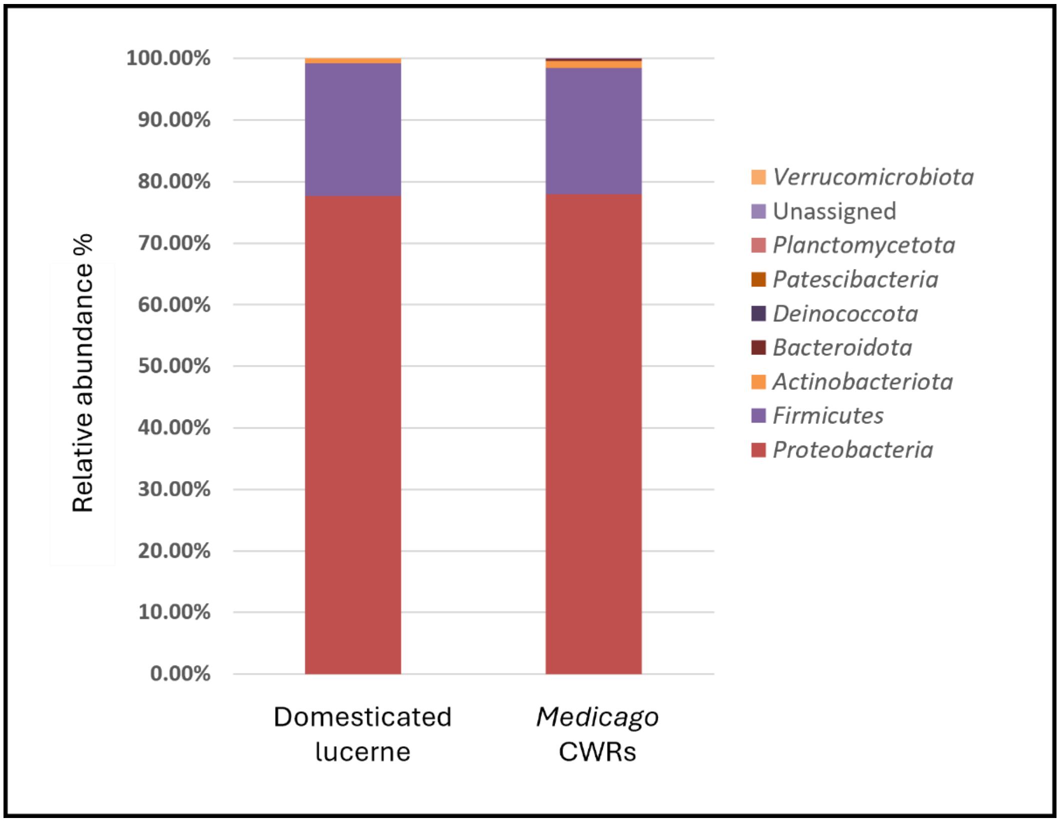


**Figure S2.** Relative ASV abundance of bacterial communities associated with *Medicago* seed microbiome at phylum level classified based on cultivation form of *Medicago* seed accessions.

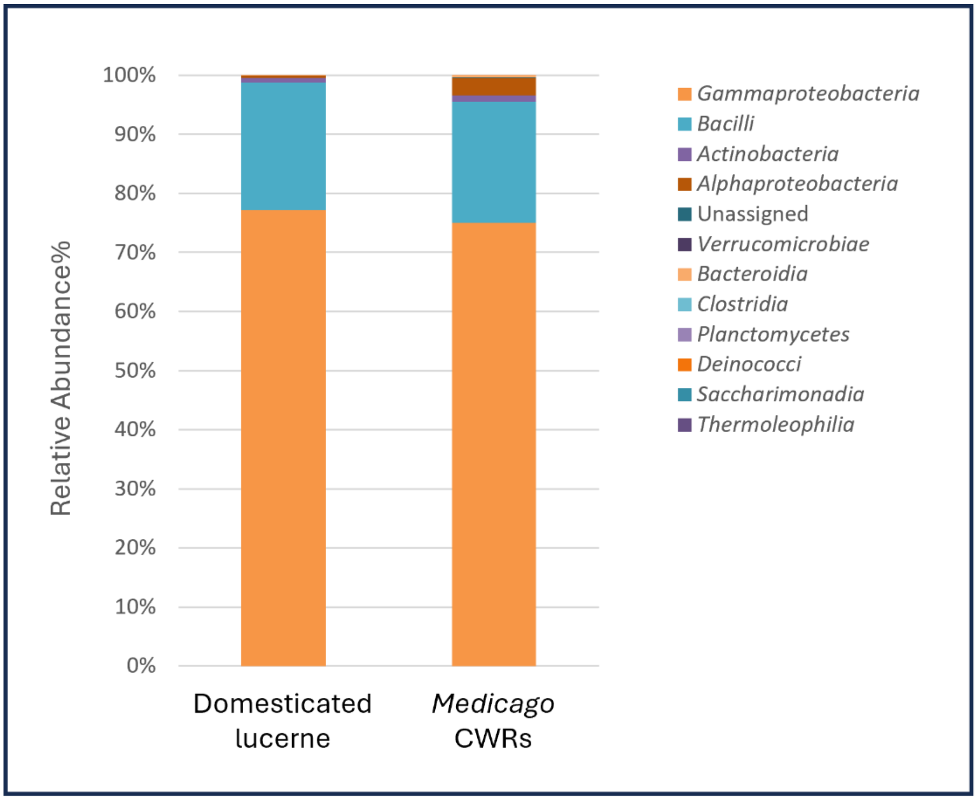


**Figure S3.** Relative OTU abundance of bacterial communities associated with *Medicago* seed microbiome at class level classified based on cultivation form of *Medicago* seed accessions

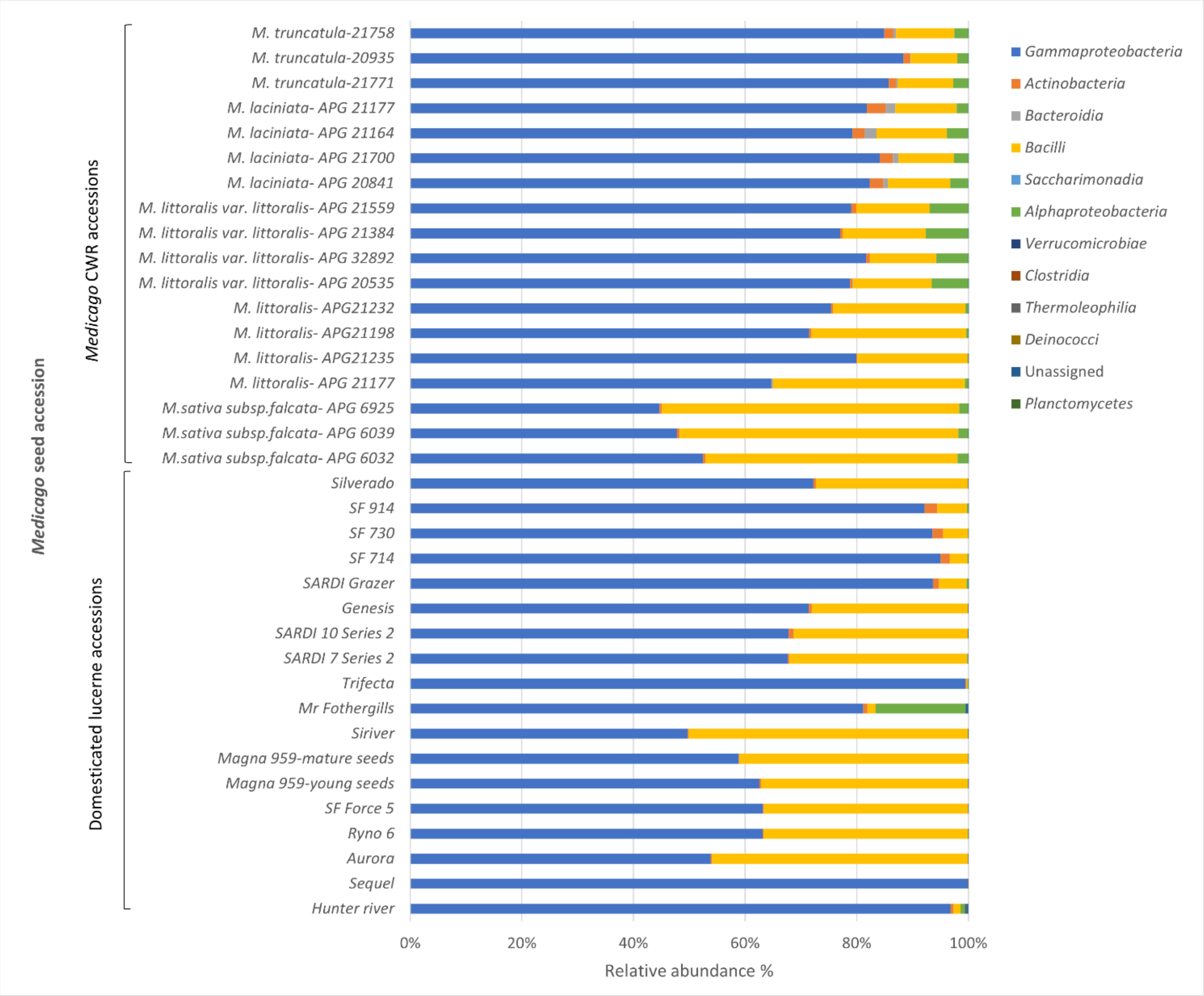


**Figure S4**. Relative ASV abundance of bacterial communities associated with *Medicago* seed microbiome at class level classified based on *Medicago* host species

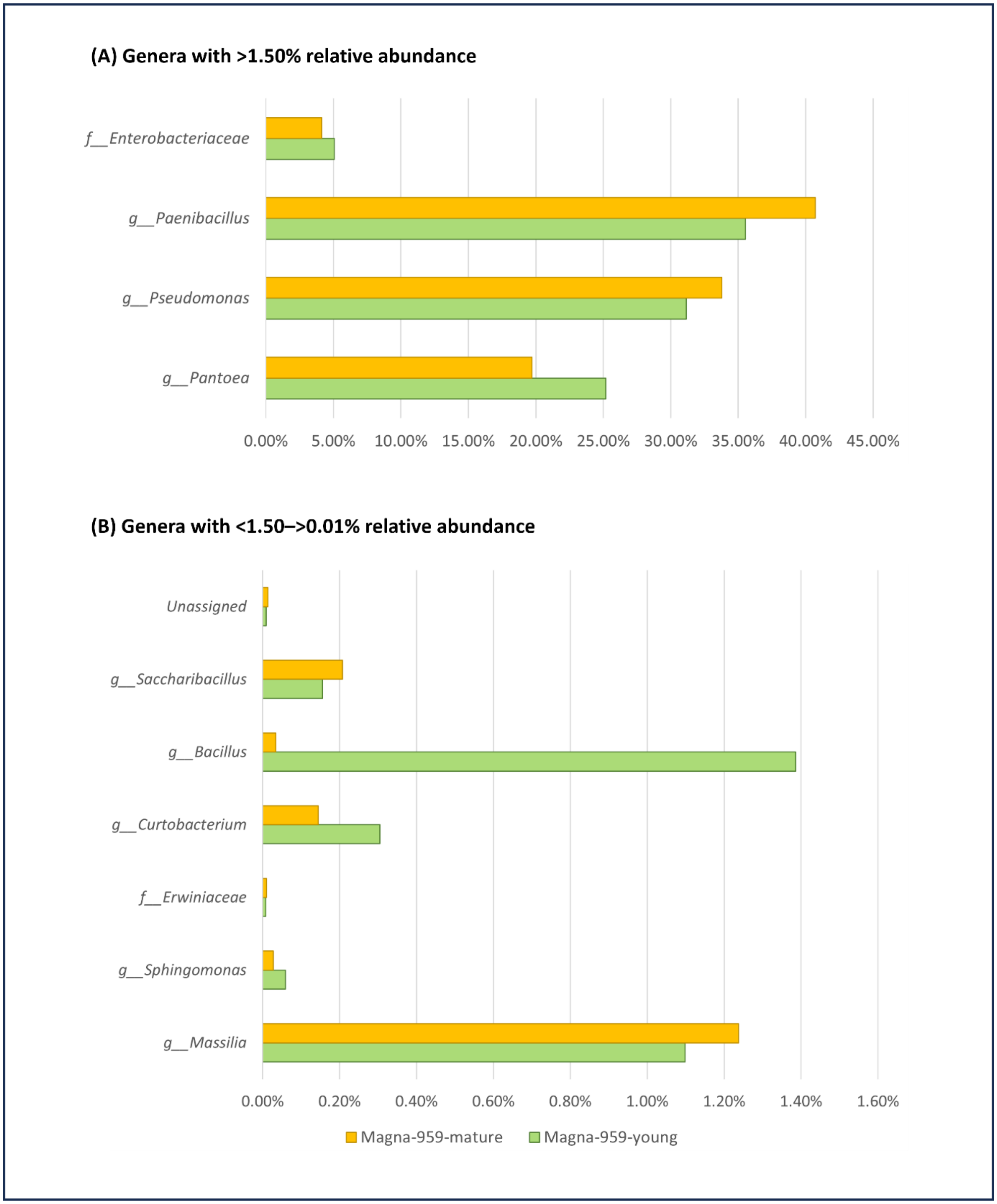


**Figure S5.** Developmental shifts in bacterial community composition in young and mature seeds of the lucerne cultivar 'Magna 959'. Bar plots show the relative abundance of bacterial taxa in Magna 959 seeds at two developmental stages: young (green) and mature (yellow). (A) Taxa with relative abundance > 1.5% in at least one condition. (B) Taxa with relative abundance between 0.01% and 1.5%, highlighting less abundant but potentially ecologically significant taxa. Taxa below 0.01% relative abundance were excluded for clarity.

**Supplementary Section S1**

DNA extraction, 16S amplicon library preparation, and sequencing

To profile bacterial communities across 36 *Medicago* cultivars, twenty-four healthy seedlings per cultivar (biological replicates) were selected. DNA was extracted from individual seedlings using the QIAGEN MagAttract 96 DNA Plant Core Kit (Qiagen®, Hilden, Germany) with minor modifications to the manufacturer's protocol for use with a Biomek® FXP lab automation workstation operated via Biomek software v4.1 and Gen5 (v2.08) (Biotek Instruments, United States).

Library preparation targeted the V4 hypervariable region (~254 bp) of the 16S rRNA gene, using a two-step PCR protocol optimised for Illumina MiSeq® sequencing (Hall and Beiko, 2018). The first PCR was performed using 515F and 806R primers tailed with Illumina adapter priming sequences. Peptide nucleic acid (PNA) PCR blockers, pPNA and mPNA, were added to suppress amplification of chloroplast and mitochondrial 16S rRNA genes, respectively (Lundberg et al., 2013). Each 25 µL PCR reaction contained 12.5 µL of KAPA HiFi HotStart 2× Ready Mix, DNA polymerase (Kapa Biosystems, London, UK), 1.25 µL of 50 µM pPNA and mPNA mix, 1.0 µL of 5 µM of each primer, 5 µL of template DNA, and PCR-grade water to volume. Thermocycling conditions were: initial denaturation at 94 °C for 3 min., 30 cycles of denaturation at 94 °C for 15 s, PNA clamping at 75 °C for 10 s, annealing at 55 °C for 10 s, and elongation at 72 °C for 45 s, followed by a final extension at 72 °C for 10 min. Amplicons were purified using ProNex® magnetic beads at a 1:1.35 (PCR product : ProNex® beads) volumetric ratio.

Index PCR was then performed using the Nextera XT Index kit (Illumina, San Diego, CA, USA). Each 50 µL reaction included 25 µL of 2× KAPA HiFi HotStart Ready Mix, 5 µL of each Nextera XT index primer 1 (N7) and 2 (S5), 5 µL of template DNA, and PCR-grade water to volume. The thermocycling program consisted of an initial denaturation at 95 °C for 3 min, 8 cycles of 95 °C for 30 s, 55 °C for 30 s, and 72 °C for 30 s, with a final extension at 72 °C for 5 min. The indexed amplicons were validated using the high sensitivity D1000 (HSD1000) ScreenTape assay on an Agilent TapeStation 2200 (Agilent Technologies, USA). Normalisation and pooling were performed using the SequalPrep™ normalisation plate (96) kit (Thermo Fisher Scientific, USA) according to the manufacturer's protocol. Equal volumes (5 µL per library) were pooled, quantified using a NanoDrop™ 2000/2000c spectrophotometer (Thermo Scientific™, USA) and a Quantus fluorometer (Promega, USA) with the QuantiFluor® dsDNA assay. Library integrity was revalidated on the TapeStation. The final library pool was denatured with 0.2 N NaOH, diluted to 6 pM, and spiked with 15% Phix control. A total of 600 µL of the prepared library was loaded onto a MiSeq reagent cartridge and sequenced on the Illumina MiSeq platform using 2 × 300 bp paired-end reads (MiSeq Reagent Kit v3), targeting the V4 regions of the 16S rRNA gene.

**Supplementary Section S2**

MiSeq data processing and analysis

Raw paired-end reads were processed using PEAR (v0.9.10) with default parameters to merge forward and reverse reads (Zhang et al., 2013). Merged reads were imported into QIIME2 **(**v2021.4) using the manifest format as single-end FASTQ files. All subsequent processing steps followed the QIIME2 “Moving Pictures” workflow (Bolyen et al., 2019). Adapter and primer sequences were trimmed using the q2-cutadapt plugin with an error rate of 0.2, and the adapter-wildcards, read-wildcards, and discard-untrimmed flags enabled. Chimeric and phiX sequences were removed using the q2-dada2 plugin. The denoise-single method was applied to generate amplicon sequence variants (ASVs) and representative sequences, with trimming low-quality regions and truncation at 253 bp based on quality profiles. Multiple sequence alignment of ASVs was performed using MAFFT (Katoh et al., 2002), and highly variable sites were masked to minimise phylogenetic noise. Phylogenetic trees (unrooted and rooted) were constructed using FastTree 2 (Price et al., 2010). Taxonomic classification was performed using the q2-feature-classifier plugin, using a naïve Bayes classifier trained on SILVA SSU database (v138), trimmed to the V4 region of the 16S rRNA gene (Bokulich et al., 2018). Low-abundance ASVs were filtered from the dataset using the q2-feature-table plugin with a minimum feature frequency of 10 and occurrence in at least two samples.

Alpha diversity was assessed using the Shannon diversity index. Initial rarefaction and diversity estimation were conducted in QIIME2, with samples subsampled to 5,061 sequences per sample. To enable enhanced visualisation and integration with experimental metadata, diversity metrics were subsequently imported into R (v4.3.1) using the qiime2R package and visualised with the phyloseq and microbiome packages. Statistical comparisons between groups were performed in QIIME2 using the Kruskal-Wallis pairwise test with false discovery rate (FDR)-corrected pairwise comparisons, which is appropriate for non-normally distributed ecological data (McKight and Najab, 2010; Willis, 2019). Beta diversity was assessed in QIIME2 using the core-metrics-phylogenetic pipeline within the q2-diversity plugin. Jaccard dissimilarity was used for ordination, while unweighted UniFrac distances were used for statistical comparisons. Phylogenetic trees were constructed using MAFFT for multiple sequence alignment and FastTree2 for approximate maximum likelihood tree building. Group differences in community composition were evaluated using permutational multivariate analysis of variance (PERMANOVA) and variability in beta dispersion was assessed using PERMDISP. Both tests were performed using QIIME2 with default parameters. A significant threshold of $p < 0.05$ was applied to all pairwise comparisons unless otherwise specified.

Core microbiome analysis was conducted in R (v4.3.1) to identify bacterial taxa present in > 90% of samples within each accession group. A binary presence–absence matrix was generated from the genus-level ASV table, and taxa exceeding the 90% prevalence threshold within each *Medicago* species were designated as core members. To visualise shared and unique components of the core microbiota, an UpSet plot was generated using the ComplexUpSet package (v1.3.3), enabling intersection analysis across all six species. The presence–absence matrix was transformed using tidyr and dplyr, and taxa were grouped by their intersection profiles. Additionally, a two-group Venn diagram comparing domesticated lucerne and CWRs was generated using the VennDiagram package (v1.7.3), based on their respective core taxa lists. All figures were formatted using ggplot2 for visual consistency.

To evaluate the culturability of dominant seed-associated taxa, an in-house BLASTn database was constructed using 16S rRNA gene sequences extracted from whole-genome assemblies of culturable bacterial isolates. Representative ASV sequences exported from QIIME2 were queried against this local database using BLASTn (NCBI BLAST+ V2.13.0), and matches with ≥ 96% sequence identity were considered culturable under the experimental conditions. This analysis enabled alignment-based comparison between culture-independent ASVs and cultured isolates, allowing estimation of the proportion of microbial community that was captured through cultivation.

## References


Bokulich, N.A., Kaehler, B.D., Rideout, J.R., Dillon, M., Bolyen, E., Knight, R., et al. (2018). Optimizing taxonomic classification of marker-gene amplicon sequences with QIIME 2's q2-feature-classifier plugin. *Microbiome* 6(1)**,** 90. doi: 10.1186/s40168-018-0470-z.

Bolyen, E., Rideout, J.R., Dillon, M.R., Bokulich, N.A., Abnet, C.C., Al-Ghalith, G.A., et al. (2019). Reproducible, interactive, scalable and extensible microbiome data science using QIIME 2. *Nat. Biotechnol.* 37(8)**,** 852–857. doi: 10.1038/s41587-019-0209-9.

Hall, M., and Beiko, R.G. (2018). "16S rRNA Gene Analysis with QIIME2," in *Microbiome Analysis: Methods and Protocols*, eds. Beiko, R.G., Hsiao, W., and Parkinson, J. (New York, NY: Springer New York), 113-129.

Katoh, K., Misawa, K., Kuma, K.i., and Miyata, T. (2002). MAFFT: a novel method for rapid multiple sequence alignment based on fast Fourier transform. *Nucleic Acids Res.* 30(14)**,** 3059-3066. doi: 10.1093/nar/gkf436.

Lundberg, D.S., Yourstone, S., Mieczkowski, P., Jones, C.D., and Dangl, J.L. (2013). Practical innovations for high-throughput amplicon sequencing. *Nat. Methods* 10(10)**,** 999-1002. doi: 10.1038/nmeth.2634.

McKight, P.E., and Najab, J. (2010). "Kruskal-Wallis Test," in *The Corsini Encyclopedia of Psychology*.), 1-1.

Price, M.N., Dehal, P.S., and Arkin, A.P. (2010). FastTree 2 – Approximately Maximum-Likelihood Trees for Large Alignments. *PLOS ONE* 5(3)**,** e9490. doi: 10.1371/journal.pone.0009490.

Willis, A.D. (2019). Rarefaction, Alpha Diversity, and Statistics. *Front. Microbiol.* 10. doi: 10.3389/fmicb.2019.02407.

Zhang, J., Kobert, K., Flouri, T., and Stamatakis, A. (2013). PEAR: a fast and accurate Illumina Paired-End reAd mergeR. *Bioinformatics* 30(5)**,** 614-620. doi: 10.1093/bioinformatics/btt593.
